# Temporal correlations among demographic parameters are ubiquitous but highly variable across species

**DOI:** 10.1111/ele.14026

**Published:** 2022-05-24

**Authors:** Rémi Fay, Sandra Hamel, Martijn van de Pol, Jean‐Michel Gaillard, Nigel G. Yoccoz, Paul Acker, Matthieu Authier, Benjamin Larue, Christie Le Coeur, Kaitlin R. Macdonald, Alex Nicol‐Harper, Christophe Barbraud, Christophe Bonenfant, Dirk H. Van Vuren, Emmanuelle Cam, Karine Delord, Marlène Gamelon, Maria Moiron, Fanie Pelletier, Jay Rotella, Celine Teplitsky, Marcel E. Visser, Caitlin P. Wells, Nathaniel T. Wheelwright, Stéphanie Jenouvrier, Bernt‐Erik Sæther

**Affiliations:** ^1^ Centre for Biodiversity Dynamics Department of Biology Norwegian University of Science and Technology Trondheim Norway; ^2^ Département de biologie Université Laval Québec City QC Canada; ^3^ College of Science and Engineering James Cook University Townsville Queensland Australia; ^4^ Department of Animal Ecology Netherlands Institute of Ecology (NIOO‐KNAW) Wageningen the Netherlands; ^5^ Laboratoire de Biométrie et Biologie Évolutive CNRS Unité Mixte de Recherche (UMR) 5558 Université Lyon 1 Université de Lyon Villeurbanne France; ^6^ Department of Arctic and Marine Biology UiT The Arctic University of Norway Tromsø Norway; ^7^ Observatoire PELAGIS UMS‐CNRS 3462 Université de la Rochelle La Rochelle France; ^8^ Département de Biologie Université de Sherbrooke Sherbrooke Québec Canada; ^9^ 6305 Department of Biosciences Centre for Ecological and Evolutionary Synthesis (CEES) University of Oslo Oslo Norway; ^10^ Department of Ecology Montana State University Bozeman Montana USA; ^11^ School of Ocean and Earth Science National Oceanography Centre University of Southampton Waterfront Campus Southampton UK; ^12^ 54998 Biology Department Woods Hole Oceanographic Institution Woods Hole Massachusetts USA; ^13^ Centre d'Etudes Biologiques de Chizé LEMAR UMR 7372 Centre National de la Recherche Scientifique Villiers en Bois France; ^14^ Department of Wildlife, Fish, and Conservation Biology University of California Davis California USA; ^15^ LEMAR CNRS IRD, Ifremer Université de Bretagne Occidentale Plouzané France; ^16^ CEFE Univ Montpellier CNRS EPHE IRD Montpellier France; ^17^ Institute of Avian Research Wilhelmshaven Germany; ^18^ Fish, Wildlife and Conservation Biology Department Colorado State University Colorado USA; ^19^ Department of Biology Bowdoin College Brunswick Maine USA

**Keywords:** capture‐recapture, demographic correlation, demography, environmental stochasticity, slow‐fast continuum, stochastic population dynamics, temporal covariation

## Abstract

Temporal correlations among demographic parameters can strongly influence population dynamics. Our empirical knowledge, however, is very limited regarding the direction and the magnitude of these correlations and how they vary among demographic parameters and species’ life histories. Here, we use long‐term demographic data from 15 bird and mammal species with contrasting pace of life to quantify correlation patterns among five key demographic parameters: juvenile and adult survival, reproductive probability, reproductive success and productivity. Correlations among demographic parameters were ubiquitous, more frequently positive than negative, but strongly differed across species. Correlations did not markedly change along the slow‐fast continuum of life histories, suggesting that they were more strongly driven by ecological than evolutionary factors. As positive temporal demographic correlations decrease the mean of the long‐run population growth rate, the common practice of ignoring temporal correlations in population models could lead to the underestimation of extinction risks in most species.

## Graphical abstract

We use long‐term demographic data from 15 bird and mammal species to quantify correlation patterns among five demographic parameters. Results show that positive correlations are ubiquitous and suggest that correlations are more strongly driven by ecological rather than evolutionary factors.

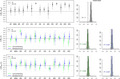

## INTRODUCTION

In an increasingly variable world, understanding stochastic population dynamics is a critical issue (Boyce et al., 2006). An important aspect of demography in stochastic environments is that population‐level demographic parameters (e.g. survival, reproduction) rarely fluctuate independently from one another but rather show temporal correlations. For instance, in good environmental conditions, survival and reproduction are often both higher than their long‐term average, whereas in poor conditions, they are often lower, which results in a positive correlation between reproduction and survival at the population level (Fay et al., 2020; Öberg et al., 2015; Reid et al., 2004). Temporal correlations between demographic parameters may amplify or alternatively attenuate the negative impact of demographic variation on population growth (Iles et al., 2019). Specifically, positive correlations should magnify the negative effect of temporal variation on population growth rate, whereas negative correlations should buffer the negative effect of demographic variation (Boyce et al., 2006; Tuljapurkar, 1982). The magnitude and direction of correlations among demographic parameters also affect elasticities, which measure the impact of a proportional change in a demographic parameter on population growth (Benton & Grant, 1996; Davison et al., 2013; Doak et al., 2005). Despite their recognized importance to our understanding of population dynamics in stochastic environments, correlations among demographic parameters have so far received little empirical interest, especially in comparison with temporal variation (Gaillard & Yoccoz, 2003; Hilde et al., 2020; Pfister, 1998).

**FIGURE 1:**
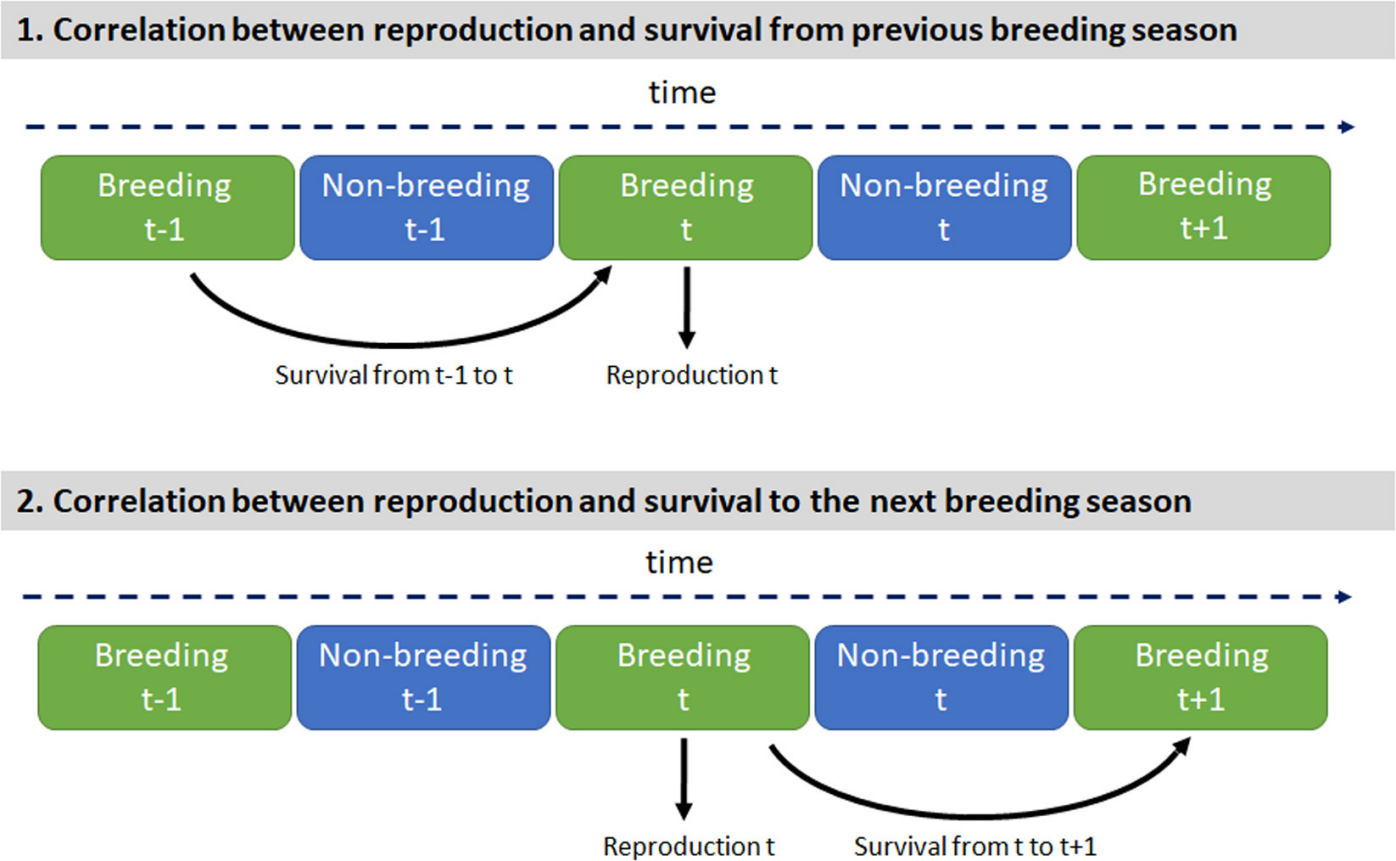
Estimation of two types of correlations between survival and reproduction. In practice, reproductive parameters are often estimated within a year, while survival probabilities between two consecutive years. This means that two types of correlations between survival and reproduction could be estimated, that is, the correlation between reproduction in a given year and either the probability of survival from the previous breeding season to the current one (i.e. survival from time t − 1 to t, type 1) or survival to the next breeding season (i.e. survival from time t to t + 1, type 2). This distinction is critical because these two types of correlations are likely to be influenced by different processes such as carryover effects of the previous non‐breeding season (causing positive correlation) versus reproductive cost carried over subsequent survival (causing negative correlation). Consequently, the direction and magnitude of the correlation likely depend on the period over which the correlation is estimated

Previous studies reported the existence of population‐level temporal correlations among demographic parameters (Jongejans et al., 2010; Reid et al., 2004; Riecke et al., 2019) and showed evidence of consequences on population dynamics (Coulson et al., 2005; Davison et al., 2013, 2019; Doak et al., 2005; Ezard et al., 2006; Wisdom et al., 2000 but see Compagnoni et al., 2016 for a weak influence). For instance, the effect of global warming on population growth of tundra plants is buffered by negative correlations between vegetative growth and both survival and reproduction (Doak & Morris, 2010). However, empirical research on temporal correlations among demographic parameters remains limited. First, most of these studies, especially those focusing on animals, were based on a single species, preventing a full understanding of how correlation patterns vary across taxa and life histories. Second, although some studies have found population‐level temporal correlations, they generally involved only a few correlations and provided little information on the direction and magnitude of these correlations, simply because this was not their primary focus (e.g. Reid et al., 2004; Sim et al., 2011). For instance, because survival is typically estimated from one breeding season to the next in vertebrate populations (i.e. pre‐ or post‐ breeding census; Caswell, 2001), correlations among survival and reproductive parameters could be assessed by considering either survival from previous (t – 1 → t) or to the next (t→t+1) breeding season (Figure 1). Surprisingly, the distinction between these two types of correlations has received little attention so far. Consequently, it is still unclear whether the sequential order between survival and reproduction has a strong effect on the correlation structure, which obscures our interpretation of the existing literature.

The identification of broad patterns of temporal correlations among demographic parameters is essential to make realistic population forecasts (Davison et al., 2013; Ferson & Burgman, 1995; Wisdom et al., 2000). When available demographic information is insufficient to estimate temporal correlations, the non‐independence among demographic parameters can be accounted for by examining a large variety of scenarios (Fieberg & Ellner, 2001). However, the uncertainty in both the direction and magnitude of demographic correlations can lead to a dramatic increase in the uncertainty of demographic inferences. In this situation, only a better understanding of correlation structures could compensate for the lack of empirical data (Fay et al., 2020). A first step toward this goal is to compare correlations estimated within pairs of demographic parameters in a standardized way across different species to assess the consistency of the direction and magnitude of the correlations. This comparative approach may allow the identification of common demographic correlation patterns across species. Furthermore, because previous studies suggest that correlations vary across species (e.g. Compagnoni et al., 2016; Jongejans et al., 2010 in plants), a second important step is, thus, to investigate factors that may predict among‐species variation. Identifying such factors would allow prediction of demographic correlations for populations for which limited information is available, such as endangered species.

A species’ position on the slow‐fast continuum of life histories is known to predict various demographic properties. For instance, species with fast life‐history strategies, characterized by an early age at maturity, high fecundity and a short lifespan, generally show larger temporal variance in demographic parameters compared with species with slower life histories that have opposite characteristics (Sæther et al., 2002,2004,2013). In addition, environmental stochasticity contributes more to variation in population growth rate in species with a faster than a slower life‐history strategy (Davison et al., 2019). This suggests that species toward the fast end of the continuum are more sensitive to variation in environmental stochasticity. Because environmental variation is a key driver of demographic correlations (Doak & Morris, 2010; Fay et al., 2020; Knops et al., 2007), species at the fast end of the continuum could thus be more prone to show correlations among demographic parameters. Therefore, for a given environmental condition, the species‐specific life history is expected to shape temporal correlations among demographic parameters, and the ranking of species along the slow‐fast continuum could be proposed as a predictor of correlation structures. Although identifying such patterns would be critical to robust extrapolation of correlation structures to unstudied species, the relationship between correlation structures in demographic parameters and species life history remains an unexplored issue. To fill this knowledge‐gap, we investigated population‐level correlations among demographic parameters in 15 bird and mammal species that are spread widely along the slow‐fast continuum of life histories. Using a multivariate normal distribution of temporal random effects implemented in a capture‐recapture modelling framework, we estimated the correlation between pairs of five demographic parameters, including juvenile survival, adult survival, reproductive probability (i.e. laying eggs in birds or giving birth in mammals), reproductive success (i.e. reproductive females successfully raising at least one offspring to fledging/weaning) and productivity (i.e. number of offspring raised per successful reproductive attempt). We addressed the following questions: (1) What is the direction, magnitude, and uncertainty of temporal correlations among demographic parameters? (2) Are correlation estimates between survival and reproduction affected by the sequential order of these events, that is, do correlations between reproduction and preceding or subsequent survival differ? (3) Are correlations among demographic parameters similar across species? (4) are correlations among demographic parameters stronger in species closer to the fast end of the slow‐fast continuum of life histories.

## MATERIAL AND METHODS

### Data sets

The accurate estimation of temporal correlations in demographic parameters requires high‐quality long‐term data sets. First, it requires individual‐based monitoring in which individuals are marked and tracked. Second, precise estimates of annual demographic parameters and temporal correlations require large sample size, with hundreds of individuals monitored over several decades (Gilljam et al., 2019; Riecke et al., 2019). These requirements strongly limit the number of data sets adequate for the investigation of temporal correlations in demographic parameters. Here, we analysed 15 high‐quality data sets from five mammal and 10 bird species (Table 1). All these populations were subjected to detailed long‐term individual monitoring ranging between 19 and 55 years, thereby satisfying the requirements for the investigation of temporal correlations. Individuals were uniquely marked at first capture and physically recaptured or resighted later in life. In this sample, generation time (i.e. mean age of mothers in a population), which reliably measures the ranking of species on the slow‐fast continuum (Gaillard et al., 2005), ranged from 1.9 (house sparrow) to 23.2 years (snow petrel), allowing for a critical investigation of variation in correlation patterns along the slow‐fast continuum of life histories (Table 1).

**TABLE 1:** Information about the population monitoring included in our analyses and the demographic parameters estimated for each species. Notation: ‘Y’ and ‘N’ indicate whether demographic parameters (juvenile survival ($\Phi_{j}$), adult survival ($\Phi_{\mathrm{ad}}$), reproductive probability (ψ), reproductive success (π) and productivity (Ω)) have been estimated for a given species. In species with fast life history, reproductive probability was not estimated because of the negligible proportion of non‐breeding individuals. Similarly, for some species with slow life history, productivity could not be estimated because they raise only one offspring at best

Species Latin name	Monitoring years	Number of individuals	Generation time	Estimated demographic parameters					Location	Ref.
				$\Phi_{j}$	$\Phi_{\mathrm{ad}}$	ψ	π	Ω		
Black‐browed albatross *Thalassarche melanophris*	1980–2015	4450	19.9	Y	Y	Y	Y	N	Kerguelen Islands, southern Indian Ocean	[1]
Bighorn sheep *Ovis canadensis*	1975–2018	520	5.8	Y	Y	Y	Y	N	Ram Mountain, Alberta, Canada	[2]
Blue tit *Cyanistes caeruleus*	1979–2018	11315	2.5	Y	Y	N	Y	Y	Corsica, France	[3]
Eurasian oystercatcher *Haematopus ostralegus*	1983–2019	2598	21.9	Y	Y	Y	Y	Y	Schiermonnikoog, Netherlands	[4]
Great tit *Parus major*	1955–2018	58234	1.8	Y	Y	N	Y	Y	Hoge Veluwe, Netherlands	[5]
Golden‐mantled ground squirrel *Callospermophilus lateralis*	1994–2019	472	2.4	Y	Y	Y	Y	Y	East River Valley, Colorado, USA	[6]
House sparrow *Passer domesticus*	1993–2013	2852	1.9	Y	Y	N	Y	Y	Hestmannøy, Norway	[7]
Kittiwake *Rissa tridactyla*	1979–2020	17280	10	Y	Y	Y	Y	Y	Cap Sizun, Brittany, France	[8]
Mountain goat *Oreamnos americanus*	1989–2018	243	7.8	Y	Y	Y	Y	N	Caw Ridge, Alberta, Canada	[9]
Roe deer *Capreolus*	1988–2020	660	5.4	Y	Y	Y	N	Y	Chizé, France	[10]
Savannah sparrow *Passerculus sandwichensis*	1986–2004	6259	2	Y	Y	N	Y	Y	Kent Island, Maryland, USA	[11]
Snow petrel *Pagodroma nivea*	1963–2017	6229	23.2	Y	Y	Y	Y	N	Terre Adélie, Antarctica	[12]
Southern fulmar *Fulmarus glacialoides*	1963–2017	1619	20.5	Y	Y	Y	Y	N	Terre Adélie, Antarctica	[13]
Weddell seal *Leptonychotes weddellii*	1986–2018	8550	16.1	Y	Y	Y	N	N	Erebus Bay, Antarctica	[14]
White‐throated dipper *Cinclus cinclus*	1978–2020	5441	2.1	Y	Y	N	Y	Y	Lyngdalselva river, Norway	[15]

Note

[1] Pardo et al., 2013 [2] Festa‐Bianchet et al., 2019 [3] Charmantier et al., 2016 [4] Van de Pol et al., 2010 [5] Visser et al., 2021 [6] Wells & Van Vuren, 2018 [7] Ranke et al., 2021 [8] Cam et al., 1998 [9] Festa‐Bianchet et al., 2019 [10] Gaillard et al., 2013 [11] Woodworth et al., 2017a [12] Barbraud & Weimerskirch, 2001 [13] Jenouvrier et al., 2003 [14] Rotella et al., 2012 [15] Gamelon et al., 2017.

### Estimating population‐level variation and covariation in demographic parameters: general model

Temporal variation and covariation in demographic parameters were estimated using a multivariate distribution within capture‐recapture models fitted in a Bayesian framework. This approach allowed us to model demographic parameters with their temporal variation and covariation within a single analysis. In addition, since the outputs of Bayesian inference are posterior distributions, it is straightforward to derive quantities while retaining uncertainties of model parameters. For example, we derived the posterior distribution of the grand mean correlation across species by iteratively averaging samples from posteriors of species‐specific correlations.

Data sets were analysed with multi‐state capture‐recapture models with the same general structure for all species. For each individual, juvenile survival (first‐year survival) was modelled as:

$${\text{Alive}}_{i,t}∼\text{Bernoulli}\left(logit^{‐1}\left(\mu_{\Phi }+\alpha_{t,\Phi ,juv}\right)\right)$$

and subsequent survival (adult survival) was modelled as:

$$\begin{array}{c} \left({\text{Alive}}_{i,t}|{\text{Alive}}_{i,t‐1}=1\right)\\ ∼\text{Bernoulli}\left(logit^{‐1}\left(\mu_{\Phi }+f_{\Phi }\left(age_{i,t}\right)+\gamma_{\Phi }*BS_{i,t‐1}+\alpha_{t,\Phi ,ad}\right)\right) \end{array}$$

where Alive*_i_*_,_ *_t_* is a dummy variable indicating whether individual *i* survived from year *t *− *1* to year *t*, $\mu_{\Phi }$ is the intercept on the logit scale, $f_{\Phi }\left({\mathrm{age}}_{i,t}\right)$ is a function of age, $\gamma_{\Phi }$ is the effect of the breeding state (BS, e.g. successful breeder vs. failed breeder) of an individual at time t‐1 on the probability of survival to year t, and $\alpha_{t,\Phi ,juv}$ and $\alpha_{t,\Phi ,ad}$ are the temporal random effects for juvenile and adult survival, respectively. Thus, we assumed that temporal variation in survival was the same for all the individuals from age one. This choice was made to ensure among‐species comparability of temporal variance and covariance. Conditional on being alive, individual *i* may breed following an additional Bernoulli process:

$$\begin{array}{c} \left({\text{Breed}}_{i,t}|{\text{Alive}}_{i,t}=1\right)\\ ∼\text{Bernoulli}\left(logit^{‐1}\left(\mu_{\psi }+f_{\psi }\left(age_{i,t}\right)+\gamma_{\psi }*BS_{i,t‐1}+\alpha_{t,\psi }\right)\right) \end{array}$$

where ${\mathrm{Breed}}_{i,t}$ is a dummy variable indicating whether individual *i* bred in year *t*, $\mu_{\psi }$ is the intercept on logit scale, $f_{\psi }\left({\mathrm{age}}_{i,t}\right)$ is a function of age, $\gamma_{\psi }$ is the effect of the breeding state and $\alpha_{t,\psi }$ is the temporal random effect. Then conditional on breeding, individual *i* may succeed in producing at least one offspring following an additional Bernoulli process:

$$\begin{array}{c} \left({\text{Success}}_{i,t}|{\text{Breed}}_{i,t}=1\right)\\ ∼\text{Bernoulli}\left(logit^{‐1}\left(\mu_{\pi }+f_{\pi }\left(age_{i,t}\right)+\gamma_{\pi }*BS_{i,t‐1}+\alpha_{t,\pi }\right)\right) \end{array}$$

where ${\mathrm{Success}}_{i,t}$ is a dummy variable indicating whether individual *i* was successful in year *t*, and all other parameters and explanatory variables have the same definitions as in the survival and reproduction model but apply to success probability (π). Finally, for species that can raise more than one offspring per year, we modelled the number of offspring produced by successful breeders (defined as productivity) as follows:

$$\begin{array}{c} \left({\text{Productivity}}_{i,t}|{\text{Success}}_{i,t}=1\right)\\ ∼\text{Distribution}\left(\text{link function}\left(\mu_{\Omega }+f_{\Omega }\left(age_{i,t}\right)+\alpha_{t,\Omega }\right)\right) \end{array}$$

Because the distribution of the number of offspring successfully raised in a given year by reproductive females strongly varied among species (Kendall & Wittmann, 2010), we chose different statistical distributions according to the average number of offspring produced. When the number of offspring produced varied little among individuals (oystercatchers (1–3), kittiwakes (1–2) and roe deer (1–3)) and few individuals produced more than one offspring, we modelled the probability of producing more than 1 offspring using a Bernoulli distribution with a logit link function. When the number of offspring produced was potentially higher, but the distribution was still skewed toward small numbers (dippers), we used a Normal distribution truncated at 0 with an identity link function. In that case, we estimated an additional parameter $\sigma_{\Omega }^{\prime 2}$, corresponding to the variation in the number of offspring produced. Finally, when the average number of offspring produced was high (blue and great tits, Savannah sparrows, ground squirrels), we used a Poisson distribution truncated at 0 with a log link function.

Temporal random effects of all demographic parameters followed a multivariate normal distribution on the scale of the link function used (i.e. logit, log or identity):

$$\left(\begin{array}{c} \alpha_{t,\Phi_{j}}\\ {\;\alpha }_{t,\Phi_{\mathrm{ad}}}\\ \begin{array}{c} {\;\alpha }_{t,\psi }\\ {\;\alpha }_{t,\pi }\\ {\;\alpha }_{t,\Omega } \end{array} \end{array}\right)\;∼\;MVN\;\left(\begin{array}{c} \begin{array}{cc} \left[\begin{array}{c} \begin{array}{c} \begin{array}{c} \;0\\ \;0 \end{array}\\ \;0 \end{array}\;\\ \begin{array}{c} \;0\;\\ \;0\; \end{array} \end{array}\right], & \left[\begin{array}{cc} \begin{array}{cc} \sigma_{\Phi_{j}}^{2} & {\mathrm{cov}}_{\Phi_{j}\Phi_{\mathrm{ad}}}\\ {\mathrm{cov}}_{\Phi_{j}\Phi_{\mathrm{ad}}} & \sigma_{\Phi_{\mathrm{ad}}}^{2}\\ \begin{array}{c} {\mathrm{cov}}_{\psi }^{1} \end{array} & \begin{array}{c} {\mathrm{cov}}_{\Phi_{\mathrm{ad}}\psi }^{1} \end{array} \end{array} & \begin{array}{ccc} {\mathrm{cov}}_{\Phi_{j}\psi }^{1} & {\mathrm{cov}}_{\Phi_{j}\pi } & {\mathrm{cov}}_{\Phi_{j}\Omega }\\ {\mathrm{cov}}_{\Phi_{\mathrm{ad}}\psi }^{1} & {\mathrm{cov}}_{\Phi_{\mathrm{ad}}\pi } & {\mathrm{cov}}_{\Phi_{\mathrm{ad}}\Omega }\\ \begin{array}{c} \sigma_{\psi }^{2} \end{array} & \begin{array}{c} {\mathrm{cov}}_{\psi \pi }^{1} \end{array} & \begin{array}{c} {\mathrm{cov}}_{\psi \Omega } \end{array} \end{array} \end{array}\right] \end{array} \end{array}\right)$$

where $\sigma_{X}^{2}$ is the variance of demographic parameter X – i.e. either juvenile survival ($\Phi_{j})$, adult survival ($\Phi_{\mathrm{ad}}$), reproductive probability (ψ), reproductive success (π) or productivity (Ω) – and $cov_{XX^{\prime }}$ is the covariance between the demographic parameters X and X'. The correlation between X and X' is calculated as: $r_{XX^{\prime }}=\frac{cov_{XX^{\prime }}}{\sigma_{X}*\sigma_{X^{\prime }}}$. Importantly, temporal random effects are shared among individuals, meaning that we estimated temporal correlation at the population‐level rather than at the individual level. The formulation of variance‐covariance among random effects shown here is for estimating the correlation between temporal effects on survival and subsequent reproduction (Figure 1). Reindexing $\alpha_{t,\Phi }$ as $\alpha_{t+1,\Phi }$ allows estimating the correlation between temporal effects on reproduction and subsequent survival (Figure 1).

Finally, detection probability was modelled as follows:

$$\begin{array}{c} \left({\text{Detection}}_{i,t}|{\text{Alive}}_{i,t}=1\right)\\ ∼\text{Bernoulli}\left(logit^{‐1}\left(\mu_{p}+f_{p}\left(age_{i,t}\right)+\gamma_{p}*BS_{i,t}+\alpha_{t,p}\right)\right) \end{array}$$

where ${\text{Detection}}_{i,t}$ indicates whether individual *i* was detected in year *t*, $\alpha_{t,p}$ is the temporal random effect assumed to be normally distributed with mean 0 and variance $\sigma_{p}^{2}$, and all other parameters and explanatory variables have the same definitions as in the survival and reproduction model but apply to detection probability (*p*).

### Species‐specific parameterization

Parameterization of the general model above was tailored for each species (Table S1). For instance, the age function for the survival probability of passerine species only included two age classes (i.e. juveniles (from fledging to age 1) vs. older individuals (≥1 year old)), whereas we distinguished four age classes in bighorn sheep (i.e. juveniles (from weaning to age 1), yearling (from age 1 to 2), prime‐age adult (from age 3 to 8), and elderly (≥9 years old)). Note that for some species we also added an interaction between age and breeding state using a pre‐breeder state for individuals from 2 years old until their first reproduction (Table S1). Importantly, although we adapted the age functions and breeding state effects on the intercept for each demographic parameter, we did not change the model structure estimating the temporal variances and correlations to make correlation estimates fully comparable across species.

While juvenile survival, adult survival and reproductive success varied over time in all species, reproductive probability was close to one in most of the short‐lived species (blue and great tit, European dipper, Savannah sparrow and house sparrow), and productivity was limited to a single offspring in most long‐lived species (Weddell seal, Antarctic fulmar, black‐browed albatross, snow petrel, mountain goat, bighorn sheep). For these constant parameters, by definition, there was no temporal variance and covariance to be estimated (Table 1).

### Relationships between temporal correlations among demographic parameters and species‐specific generation time

To assess the relationship between demographic correlations and the species’ pace of life, we regressed species‐specific mean correlation estimates against their generation time. Generation time is defined as the mean age of females (in years) when they lay eggs or give birth to offspring and was obtained from age‐structured population projection models parametrized with the average demographic parameters estimated. Generation time was computed as the inverse of the sum of the elasticities of the growth rate to changes in fecundities (Bienvenu & Legendre, 2015). Due to the relatively small number of species, we did not correct for phylogenetic relatedness among species (see Sæther et al., 2013 for a similar argument).

### Model implementation

We used a Bayesian approach for inference on the model parameters, relying on Markov chain Monte Carlo (MCMC) methods for posterior sampling. We conducted the analyses in JAGS (Plummer, 2003) via the R package jagsUI (Kellner, 2016, see Appendix S1 for an example of JAGS code used). We modelled the variance‐covariance matrix using the Cholesky decomposition with Parameter Expansion following Chen and Dunson (2003, see Appendix S2 for details). We carried out a prior sensitivity analysis to assess the effect of prior choice on correlation estimates (Appendix S3). Results show that prior choice is unlikely to have affected our results (Figure S1). Posterior summaries were based on 3000 values extracted from three or four Markov chains. The number of iterations (range 3000–30,000), burn‐in (range 1000–5000), and thinning intervals (1–25) varied among species according to the difficulty in reaching convergence. We confirmed convergence of MCMCs for each parameter by graphical examination and using the Gelman Rubin statistic (satisfied with all R‐hat ≤ 1.1, Brooks & Gelman, 1998). To gauge the evidence of an effect, we calculated the proportion of the posterior distribution that had the same sign as the posterior mean, ‘*P*’. Values of *P* that are close to 1 indicated strong evidence of an effect with a given sign, while values close to the minimum value of 0.5 indicated no clear evidence.

## RESULTS

### What is the pattern of temporal correlations among demographic parameters?

**FIGURE 2:**
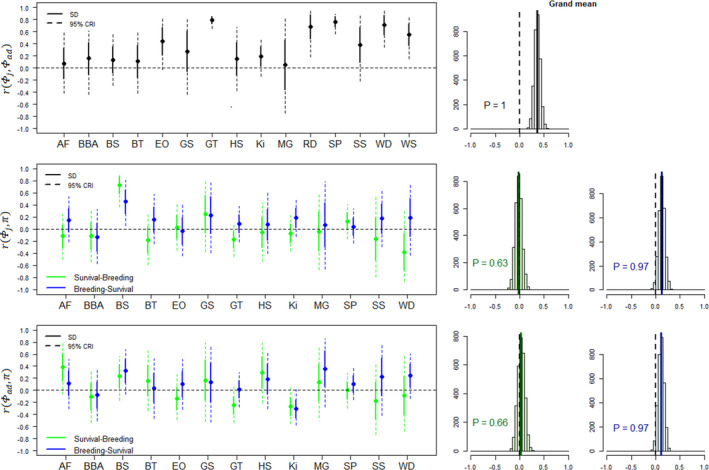
Temporal correlations estimated for three pairs of demographic parameters including juvenile survival ($\Phi_{j}$), adult survival ($\Phi_{\mathrm{ad}}$) and reproductive success (π). We estimated correlations between reproductive success and both survival from the previous and to the next reproductive season, leading to two correlation estimates (green and blue, respectively). Species names: AF = Antarctic fulmar, BBA = black‐browed albatross, BS = bighorn sheep, BT = blue tit, EO = Eurasian oystercatcher, GS = golden‐mantled ground squirrel, GT = great tit, HS = house sparrow, Ki = kittiwake, MG = mountain goat, RD = roe deer, SP = snow petrel, SS = Savannah sparrow, WD = white‐throated dipper, WS = Weddell seal. For notation, ‘SD’ indicates standard deviation, ‘CRI’ indicates credible interval and ‘P’ indicates the proportion of the posterior distribution that has the same sign as the posterior mean

**FIGURE 3:**
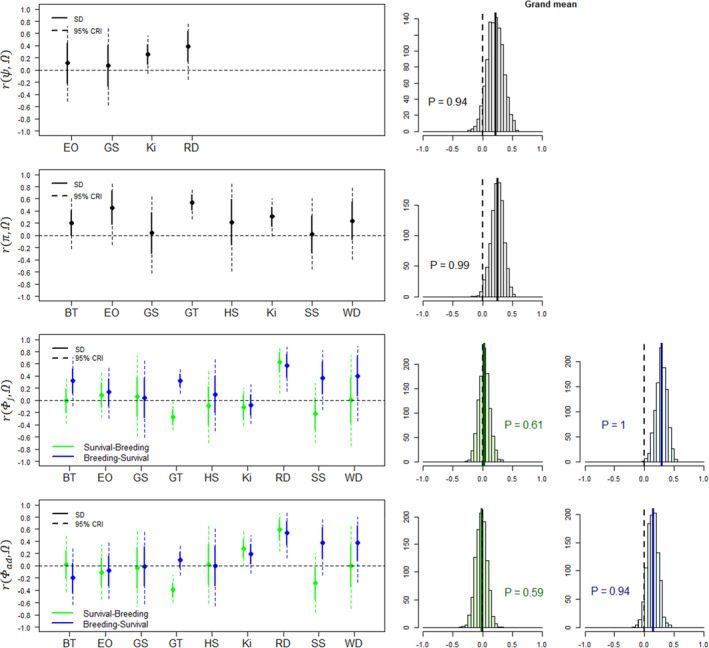
Temporal correlations estimated between productivity (Ω) and four demographic parameters, including juvenile survival ($\Phi_{j}$), adult survival ($\Phi_{\mathrm{ad}}$), breeding probability (ψ) and reproductive success (π). We estimated correlation between productivity and both survival from the previous and to the next reproductive season, leading to two correlation estimates (green and blue, respectively). Species names: BT = blue tit, EO = Eurasian oystercatcher, GS = golden‐mantled ground squirrel, GT = great tit, HS = house sparrow, Ki = kittiwake, RD = roe deer, SS = Savannah sparrow, WD = white‐throated dipper. For notation, ‘SD’ indicates standard deviation, ‘CRI’ indicates credible interval and ‘P’ indicates the proportion of the posterior distribution that has the same sign as the posterior mean

**FIGURE 4:**
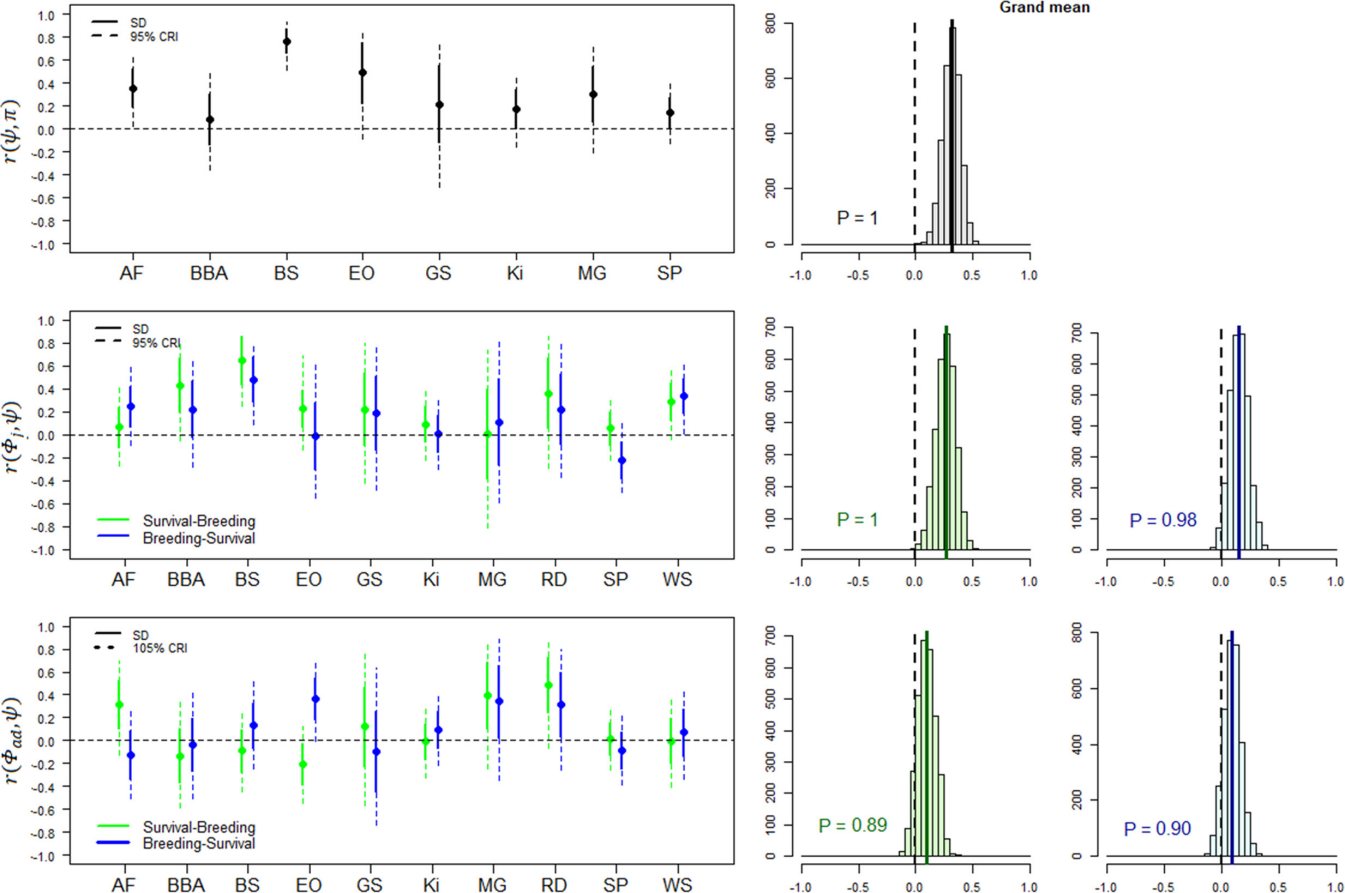
Temporal correlations estimated for three pairs of demographic parameters including juvenile survival ($\Phi_{j}$), adult survival ($\Phi_{\mathrm{ad}}$), reproductive probability (ψ) and reproductive success (π). We estimated correlations between reproductive performances and both survival from the previous and to the next reproductive season, leading to two correlation estimates (green and blue, respectively). Species names: AF = Antarctic fulmar, BBA = black‐browed albatross, BS = bighorn sheep, GS = golden‐mantled ground squirrel, Ki = kittiwake, MG = mountain goat, Oy = Eurasian oystercatcher, SP = snow petrel, WS = Weddell seal. For notation, ‘SD’ indicates standard deviation, ‘CRI’ indicates credible interval and ‘P’ indicates the proportion of the posterior distribution that has the same sign as the posterior mean

Grand mean correlations among demographic parameters across species were moderate, with posterior means ranging from −0.02 for correlations between productivity and adult survival to the next reproductive season to 0.36 for correlations between juvenile and adult survival. Grand mean correlations were more frequently positive than negative, with estimates (i.e. posterior means) being positive in 14 out of 16 cases (Figures 2, 3, 4). These positive grand mean correlations ranged from 0.02 to 0.36 and received strong support in ten cases (*P* ≥ 0.94). The estimates of these ten correlations were also more frequently positive at the species level (91%, *n* = 96, Figures 2, 3, 4). In contrast, the two grand mean correlations with negative estimate ranged from −0.01 to −0.02, and both received very weak support (*P* < 0.64). Furthermore, correlation estimates within species were not consistently negative for these two correlations since only 56% of species‐specific correlations were negative (*n* = 18). Overall, species‐specific correlations were very uncertain: 137 out of 158 (87%) of the species‐specific correlation estimates showed 95% credible intervals (CRI) that overlapped with zero (Table S2).

### Does sequential order affect the correlation between survival and reproduction?

The sequential order chosen to estimate the correlation between reproduction and survival (i.e. considering survival from vs. to a given reproductive season, Figure 1), had a strong impact on the estimates (Figures 2, 3, 4). Overall, posterior means of correlations were more frequently negative when correlations were assessed between reproduction and survival from the previous reproductive season compared with survival from current breeding season to the next (42% vs. 21%, *n* = 62). The effect of the reproduction‐survival sequential order on correlations also varied according to the species. For some species (e.g. great tit, Savannah sparrow, southern fulmar), estimates changed according to the type of correlation that was modelled, affecting both the magnitude and direction of correlations, but for others (e.g. black‐browed albatross, ground squirrel, mountain goat), estimates were similar. For example, for Savannah sparrow, posterior mean of the correlation between productivity and adult survival to the next reproductive season was 0.37 but changed to −0.29 for survival from the previous reproductive season, while they were both close to 0 for house sparrow. Changes in the direction of the posterior mean of the correlation between reproduction and survival according to when survival was measured were quite common, occurring in 43% of the estimated correlations (*n* = 62). Nevertheless, these shifts were uncertain for most species. Posterior distributions of the difference between pairs of correlations frequently crossed 0.

### Are correlations among demographic parameters similar across species?

Across species, the consistency of the direction of correlations varied depending on the focal pair of demographic parameters. For instance, although correlations between juvenile and adult survival and between reproductive probability and reproductive success were consistently positive across species (Figures 2 and 4), the direction of the correlation between reproductive success and adult survival was more variable, regardless of when survival was measured (Figure 2). Posterior means of correlations were generally highly variable among species for all pairs of demographic parameters (SD = 0.22). For instance, even though the posterior means of correlations between juvenile and adult survival were positive in all species, the magnitude of the correlation varied a lot among species, with estimates ranging from 0.08 (southern fulmar) to 0.77 (snow petrel) (Figure 2; Table S2).

### Are correlations among demographic parameters stronger in faster species?

**FIGURE 5:**
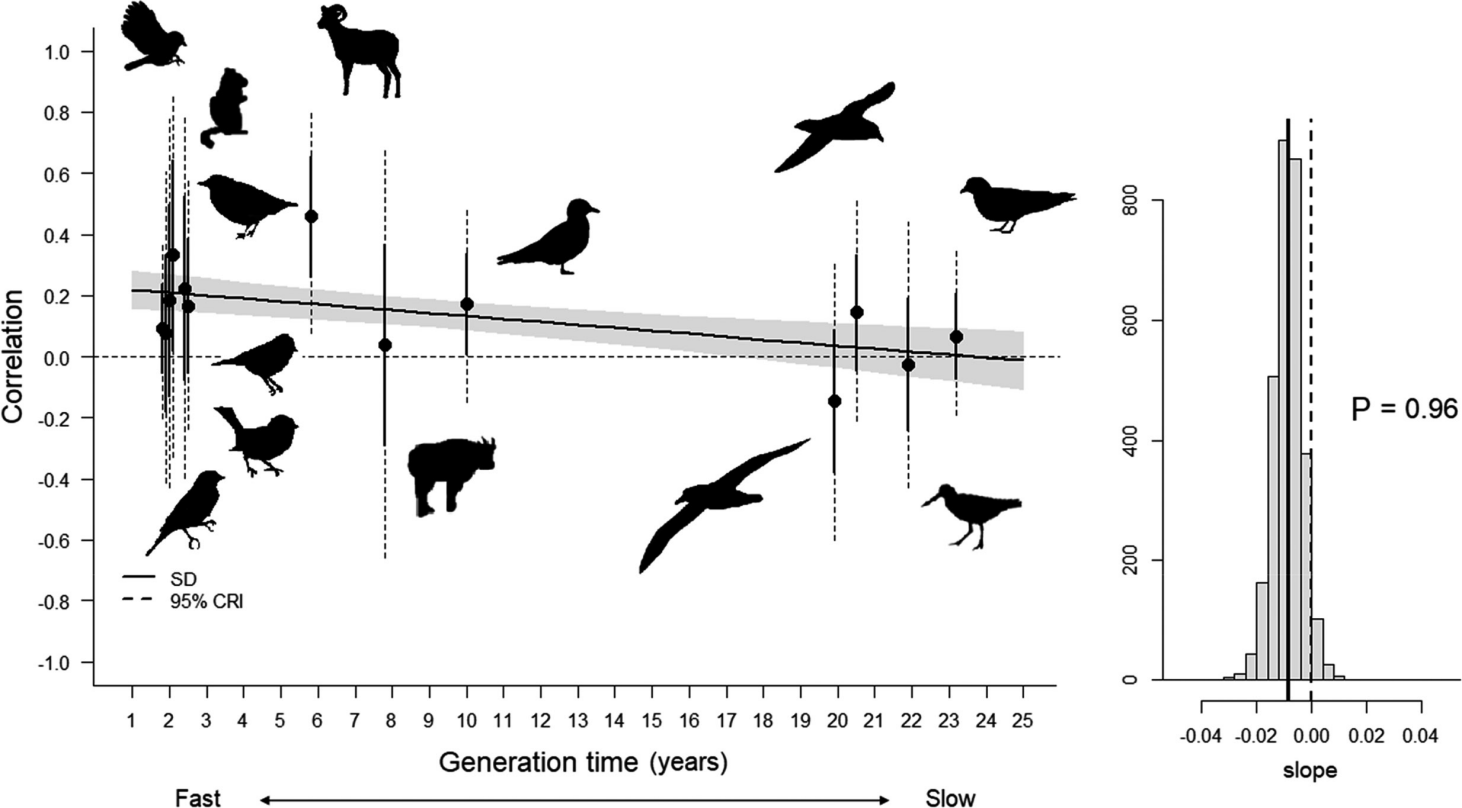
Temporal correlation between reproductive success and subsequent juvenile survival as a function of generation time. ‘P’ indicates the proportion of the posterior distribution that has the same sign as the posterior mean

Among‐species variance in correlation was poorly explained by species generation time, which accounted for <10% of the variation observed among species‐specific correlations for 11 correlations out of 15 (Table S3). Furthermore, support for a relationship between temporal correlations and generation time was weak in all cases (*P * < 0.89, Table S3) except for the negative relationship between juvenile survival and reproductive success and generation time (slope = −0.008, *P* = 0.96, Figure 5). This correlation varied in the a priori predicted direction from ca. 0 for species with a slow pace of life (generation time >15 years) to ca. 0.20 for species with a fast pace of life (generation time <7 years, Figure 5).

## DISCUSSION

Identification of broad patterns of temporal correlations among demographic parameters is essential for our understanding of population dynamics in variable environments but has not been yet thoroughly investigated, especially in animals. We filled this knowledge gap by investigating correlations among five demographic parameters across 15 bird and mammal species with contrasting life histories. Overall, we found that correlations among demographic parameters are ubiquitous, more frequently positive than negative, but that their magnitude is highly variable among species and difficult to predict based on species‐specific life history. Here, we discuss the various ecological and evolutionary mechanisms from which this pattern could result, and conclude that correlations among demographic parameters are most likely driven by the environmental context.

### Positive correlations are ubiquitous

Positive correlations were clearly more prominent than negative correlations. This finding is consistent with previous studies and supports that positive correlations among demographic parameters are the rule rather than the exception across species (Coulson et al., 2005; Ezard et al., 2006; Fay et al., 2020; Jongejans et al., 2010; Morris et al., 2011; Reid et al., 2004; Sæther & Bakke, 2000). This suggests that environmental stochasticity generally affects demographic parameters in the same way, generating years with good conditions where most demographic parameters are higher than their long‐term average, and years with poor conditions where most demographic parameters are lower than their long‐term average. Among environmental factors, climatic conditions and food availability are expected to be key factors generating positive correlations (Fay et al., 2020; Paniw et al., 2020). For instance, the strong positive correlation between juvenile survival and adult survival found here for great tits is likely due to variation in availability of winter food resources that drives the annual survival of both juvenile and adult individuals in this population (Perdeck et al., 2000). In long‐lived seabirds, years with high reproductive probability were also years with high reproductive success likely because both are driven by climatic conditions that affect foraging condition and/or food availability (Jenouvrier et al., 2015, 2018; Sauser et al., 2021). In addition, for seabirds, climatic conditions affecting reproduction can affect juvenile survival, for instance through their impact on fledging condition, hence generating a positive covariation between these traits (Jenouvrier et al., 2015; Sauser et al., 2018).

This general pattern of positive correlations has important implications for population management and conservation. Overall, positive correlations tend to destabilize population dynamics by decreasing the mean and increasing the variance of the long‐run population growth rate and thereby increasing extinction risk. Thus, the increase in environmental variation predicted under ongoing global climate change (Masson‐Delmotte et al., 2021) is likely to negatively affect population growth through an increase of both variance and covariance in and among demographic parameters. The predominance of positive correlations makes their inclusion into population models critical since ignoring them would lead to overoptimistic population forecasts. Yet, most conservation studies relying on demographic models still ignore temporal correlations among demographic parameters (e.g. >80% in the review from Earl, 2019).

### Among‐species variation in correlation structure

We found high among‐species variation in the magnitude of the correlations observed for a given pair of demographic parameters. This variation is also supported by previous studies comparing demographic correlations among plant species (Compagnoni et al., 2016; Jongejans et al., 2010). Contrary to our expectation, among‐species variation was poorly predicted by generation time. This contrasts with previous research conducted on temporal variance. Indeed, the demographic buffering hypothesis predicts that traits that have the highest potential impact on population growth rate should be the most buffered against environmental variation. As the potential influence of demographic parameters on population growth rate is a direct function of generation time (Hamilton, 1966), temporal variation in demographic parameters is also expected to vary along the slow‐fast continuum (as reported by Barraquand et al., 2014; Gaillard & Yoccoz, 2003; Hilde et al., 2020). The weak empirical evidence we report for the decrease of demographic correlations with generation time (only 1 out of 15 comparisons supported this prediction) shows that these relationships are unlikely to be general in nature.

Weak relationships between generation time and temporal correlations suggest that temporal correlations are primarily driven by ecological factors (e.g. climatic conditions, food availability, predation pressure) rather than among‐species variation in life histories. For instance, although Savannah sparrows and Eurasian oystercatchers display very different life‐history strategies, located close to either end of the slow‐fast continuum (i.e. generation time of 2 and 22.5 years, respectively), both show a similar positive correlation between juvenile survival and adult survival, most likely because of the critical role of winter temperature in determining survival of all individuals in both species (van de Pol et al., 2010; Woodworth et al., 2017b). Because the ecological context is much more variable than life‐history strategies, demographic correlations are likely to be population‐specific. Making accurate predictions about the direction and magnitude of temporal correlations may, thus, require a detailed understanding of species ecology and a good knowledge of environmental factors driving population dynamics.

### Effects of trade‐offs and density dependence

Although environmental stochasticity is expected to be the key process generating population‐level covariation among demographic parameters, other processes such as life‐history trade‐offs and density dependence could also play a role. Energy allocation trade‐offs generate non‐independent variation in demographic parameters at the individual level that may scale up to the population level to generate negative temporal covariation among demographic rates (Van Tienderen, 1995). For instance, trade‐offs between growth and reproduction at the individual level can generate a negative temporal correlation between these traits at the population level in some plants (e.g. Compagnoni et al., 2016). Nevertheless, this scaling up is expected to occur only when variation in resource acquisition is smaller than variation in resource allocation (Descamps et al., 2016; van Noordwijk & de Jong, 1986). In most cases, empirical studies typically report positive rather than negative correlations between traits competing for the same resources (e.g. growth and reproduction in plants (Jongejans et al., 2010), survival and reproduction in animals (Coulson et al., 2005; Fay et al., 2020; Morris et al., 2011)). This suggests that trade‐offs are often masked and dominated by environmental stochasticity, and that demographic correlations are primarily driven by the absolute amount of resources available in the environment.

Density dependence may also affect temporal correlations among demographic parameters either directly, via short‐term density feedback, or indirectly by modulating the effect of environmental stochasticity. Direct effects can take place when density dependence happens within a short period (i.e. a year). For instance, strong winter mortality may allow higher breeding probability the following breeding season because of the lower population density (Pradel et al., 1997; Wauters et al., 2004). Such short‐term density feedback could explain why the correlations between reproductive performance and survival shift from negative to positive within some species depending on whether one considers survival from or survival to this attempt. In Savannah sparrows, for instance, although annual survival is mostly density‐independent and thus weakly affected by the number of new individuals produced, survival from the previous reproductive season strongly influences reproductive parameters because the breeding performance is under strong density‐dependence (Woodworth et al., 2017a, 2017b). When reproduction is mostly affected by density dependent factors and survival by density‐independent factors (e.g. environmental stochasticity), both a negative correlation between reproductive performance and survival from the previous reproductive season and a positive correlation between reproductive performance and survival to the next breeding season may co‐occur. Accordingly, after accounting for population density (Appendix S4), grand mean correlations were all positive or null, including correlations between reproduction and survival from the previous breeding season (Figures S2, S3, S4 & S5).

Indirect density dependence could be equally common. High population density is expected to magnify the effect of poor environmental conditions, while low population density may decrease the negative effect of poor conditions (Barbraud & Weimerskirch, 2003; Coulson et al., 2001; Sandvig et al., 2017). For instance, both juvenile survival and reproductive performance in bighorn sheep are positively affected by precipitation, but this effect is magnified at high density (Portier et al., 1998). This interplay between population density and environmental conditions is likely shaping the strong correlation between juvenile survival and reproductive performance (both reproductive probability and reproductive success) observed in this population. Similarly, high population density is known to magnify the negative effect of environmental stochasticity on demographic parameters in the dipper (Gamelon et al., 2017).

### Challenges when estimating correlations

Although the data sets we analysed are among the most comprehensive individual‐based long‐term monitoring available, uncertainty in correlation estimates was large and most of them had 95% CRI overlapping 0. This considerable uncertainty associated with correlation estimates has also been reported in previous studies (Compagnoni et al., 2016; Fay et al., 2020) and shows that precise estimation of temporal correlations is challenging and requires large sample size (Gilljam et al., 2019; Riecke et al., 2019). It is therefore unsurprising that many studies did not detect any correlation among demographic parameters (Jongejans et al., 2010), but this does not necessarily mean that correlations are non‐existent or even negligible. While correlation estimates are uncertain, they often show a consistent positive pattern across species. Such consistency in the direction of the correlation would not be expected if true correlations were null and observed magnitude simply an artefact of sampling variance. Consequently, ignoring correlations for which 95% CRI overlap with zero would lead one to assume in many circumstances that demographic parameters are independent while they are actually correlated (with potential implications for population growth rate Boyce et al., 2006; Tuljapurkar, 1982). Although strong correlations can be detected in very small data sets (Ramula & Lehtilä, 2005; type M error *sensu* Gelman & Carlin, 2014), the absolute effect sizes of demographic correlations are generally underestimated (Fay et al., 2021; Riecke et al., 2019). Indeed, sampling variance increases raw variance and decreases raw covariance, leading to the underestimation of the correlation since $cor_{\mathrm{AB}}=cov\left(A,B\right)/\left(\sigma_{A}\times \sigma_{B}\right)$. Critically, even if the magnitude of correlations is frequently underestimated and very uncertain, the direction of the correlation is generally well estimated and could provide useful information about correlation patterns (Fay et al., 2021; Riecke et al., 2019).

## CONCLUSION

Although temporal correlations among demographic parameters are challenging to estimate precisely, even from some of the longest‐running vertebrate studies in the world, we stress the need to incorporate them routinely in population models. Positive correlations are ubiquitous and ignoring these positive correlations would lead to overoptimistic population forecasts, especially for small populations in which density dependence is weak. Our results indicate that correlations are more strongly driven by ecological rather than evolutionary factors. This makes the anticipation of correlations challenging for species for which little information is available because the population ecological context appears to matter most. However, the average effect sizes we reported provide realistic values that should be useful to simulate reliable population forecast in birds and mammals. Furthermore, in systems where the main environmental drivers have been identified and affects simultaneously several vital rates, including these drivers in population models could account for most of the non‐independence of the temporal variation in vital rates.

## CONFLICTS OF INTEREST

No conflict of interest to declare.

## AUTHOR CONTRIBUTIONS

RF conceived the ideas for this paper in collaboration with BS, JMG, MP, SH, SJ and NGY. CB, CB, DV, EC, KD, MG, MM, FP, JR, CT, MV, CW and NT supervised the long‐term data collection. RF performed the modelling and analyses with the help of ANH, BL, CL, KM, MA and PA. RF interpreted the results with the help of BS, JMG, SJ and NGY. RF wrote the paper with feedback and editing from all co‐authors.

### PEER REVIEW

The peer review history for this article is available at https://publons.com/publon/10.1111/ele.14026.

## Supporting information

Supplementary Material

## Data Availability

Should the manuscript be accepted, the data supporting the results will be archived on a Dryad repository: https://doi.org/10.5061/dryad.r2280gbfq.
