# Supplementary material for: Temporal correlations among demographic parameters are ubiquitous but highly variable across species
Source: Ecol Lett. 2022 May 24;25(7):1640–54. doi: 10.1111/ele.14026 (PMC9323452; doi:10.1111/ele.14026)
Supplement: Supplementary file 1 — Supplementary Material [file ELE-25-1640-s001.docx]

**Table S1: Age and state dependence parametrization**

Note that for some species we included an interaction between age and breeding state using a pre-breeder state that represents individuals from 2 years-old until their first reproduction. This pre-breeder state is useful to describe the life cycle of long-lived species for which the age at first reproduction can be delayed by several years. The pre-breeder state allows modelling of the progressive recruitment of immatures into the breeding part of the population.

Notation: demographic parameters include survival ($\Phi$), reproductive probability (ψ), reproductive success (π) and productivity (Ω). “,” and “=” indicate that age classes or state effect were separated and grouped (equal), respectively. For age structure, 1 indicates age one, 2 age two and so on. For survival, age 1 corresponds to the survival between age 0 (fledgling) and age 1 (one-year old). “+” indicates that older age classes were pooled. “PreB” and “Ad” indicate that we distinguished pre-breeders (from 2 years-old until the age of first reproduction), and adult mature individuals (recruited individuals). For state dependence, “NB” indicates non-breeder, “FB” indicates failed breeder, “SB” indicates successful breeder and “FirstB” indicates first-time breeder. “NA” indicates that this demographic parameter was not modelled for this species. “-“ indicates that we did not define age or state dependence, i.e., we used a single constant parameter. Species names: AF = Antarctic fulmar, BBA = black-browed albatross, BS = bighorn sheep, BT = blue tit, EO = Eurasian oystercatcher, GS = golden-mantled ground squirrel, GT = great tit, HS = house sparrow, Ki = kittiwake, MG = mountain goat, SP = snow petrel, SS = Savannah sparrow, WD = white-throated dipper, WS = Weddell seal.

| **Species** | **𝛷** | **ψ** | **π** | **Ω** | **p** |
| --- | --- | --- | --- | --- | --- |
|  | **Age structure** | | | | |
| **AF** | 1,PreB,Ad | 1=5,6,7,8,9,10,11,12+  Ad: - | - | NA | 1=3,4,5,6,7,8,9,10+ |
| **BBA** | 1,PreB,Ad | PreB:1=3,4,5,6,7,8,9,10+  Ad: - | - | NA | PreB:1,2,3,4,5,6,7,8+  Ad: - |
| **BS** | 1,2,3=8,9+ | PreB:1,2,3,4+  Ad:2,3,4=12,13+ | 1,2,3,4=12,13+ | NA | - |
| **BT** | 1,2+ | NA | - | 1,2+ | - |
| **EO** | 1,PreB,Ad | PreB:1,2=3,4,5,6,7,8+  Ad: - | - | - | 1,2,3+ |
| **GS** | 1,2+ | PreB:1,2+  Ad: - | 1,2+ | 1,2+ | - |
| **GT** | 1,2+ | NA | - | 1,2+ | - |
| **HS** | 1,2+ | NA | - | - | - |
| **Ki** | 1,2,3,4,5+ | PreB:1,2,3,4,5+  Ad: - | - | - | PreB:1,2,3,4,5+  Ad: - |
| **MG** | 1,2,3=8,9+ | PreB:1,2,3,4,5+  Ad:3=6,7=9,10+ | 1,2,3=6,7=9,10+ | NA | - |
| **RD** | 1,2,3=11,12+ | 1,2=11,12=13,14+ | NA | 1,2,3=4,5=8,9=12,13+ | - |
| **SP** | 1,PreB,Ad | PreB:1=4,5,6,7,8,9,10,11+  Ad: - | - | NA | PreB:1=3,4,5,6,7,8,9,10,11,12,13,14,15 Ad: - |
| **SS** | 1,2+ | NA | 1,2+ | 1,2+ | - |
| **WD** | 1,2+ | NA | 1,2+ | 1,2+ | 1,2+ |
| **WS** | 1,PreB(2,3,4+),Ad | PreB:1=3,4,5,6,7,8,9,10,11,12+ Ad: - | NA | NA | PreB:1,2,3=6,7+  Ad: - |
|  | **State dependence structure** | | | | |
| **AF** | NB, FB, SB | NB, FB, SB | NB, FB, SB | NA | NB, FB, SB |
| **BBA** | NB, FB, SB | NB, FB, SB | FirstB,NB, FB, SB | NA | NB, FB, SB |
| **BS** | NB, FB=SB | NB, FB=SB | NB, FB=SB | NA | - |
| **BT** | - | NA | - | - | - |
| **EO** | - | NB, FB=SB | - | - | NB, FB=SB |
| **GS** | - | - | - | - | - |
| **GT** | - | NA | - | - | - |
| **HS** | - | NA | - | - | - |
| **Ki** | NB, FirstB, FB, SB | NB, FB, SB | NB, FirstB ,FB, SB | NB, FirstB, FB, SB | - |
| **MG** | NB, FB=SB | NB, FB=SB | NB, FB=SB | NA | - |
| **RD** | - | - | - | - | - |
| **SP** | NB=FB,SB | NB, FB=SB | NB, FB=SB | NA | NB, FB, SB |
| **SS** | - | NA | - | - | - |
| **WD** | - | NA | - | - | - |
| **WS** | NB, FirstB, FB=SB | NB, FirstB, FB=SB | NA | NA | NB, FB=SB |

**Table S2: Species-specific estimates of temporal correlations among demographic parameters.** For the correlation between survival and reproduction, we can estimate two types of correlation (Fig. 1). Type 1 corresponds to the correlation between reproductive performance and survival from the previous breeding season, while type 2 corresponds to the correlation between reproduction and survival to the next breeding season. Estimations provided include the posterior mean and 95% credible interval. Notation: $\Phi_{j}$ = juvenile survival, $\Phi_{ad}$ = adult survival, ψ = reproductive probability, π = reproductive success, Ω = productivity, AF = Antarctic fulmar, BBA = black-browed albatross, BS = bighorn sheep, BT = blue tit, EO = Eurasian oystercatcher, GS = golden-mantled ground squirrel, GT = great tit, HS = house sparrow, Ki = kittiwake, MG = mountain goat, SP = snow petrel, SS = Savannah sparrow, WD = white-throated dipper, WS = Weddell seal.

| **Correlation pair** | | | | |
| --- | --- | --- | --- | --- |
| **Species** | $r_{\Phi_{j}\Phi_{ad}}$ | $r_{\psi\pi}^{1}$ | $r_{\pi\Omega}^{1}$ | $r_{\psi\Omega}^{1}$ |
| **AF** | 0.08 [-0.42, 0.67] | 0.36 [0.02, 0.65] | NA | NA |
| **BBA** | 0.16 [-0.44,0.61] | 0.08 [-0.36, 0.51] | NA | NA |
| **BS** | 0.13 [-0.29, 0.55] | 0.77 [0.51, 0.93] | NA | NA |
| **BT** | 0.11 [-0.42, 0.62] | NA | 0.20 [-0.23, 0.60] | NA |
| **EO** | 0.44 [-0.03, 0.84] | 0.49 [-0.09, 0.88] | 0.45 [-0.16, 0.85] |  |
| **GS** | 0.28 [-0.44, 0.84] | 0.21 [-0.50, 0.77] | 0.04 [-0.62, 0.66] |  |
| **GT** | 0.79 [0.66, 0.89] | NA | 0.55 [0.27, 0.76] | NA |
| **HS** | 0.15 [-0.38, 0.67] | NA | 0.22 [-0.55, 0.86] | NA |
| **Ki** | 0.19 [-0.13, 0.50] | 0.17 [-0.15, 0.48] | NA |  |
| **MG** | 0.02 [-0.74, 0.75] | 0.31 [-0.19, 0.75] | NA | NA |
| **RD** | 0.68 [0.20,0.94] | NA | NA | 0.39 [-0.16,0.80] |
| **SP** | 0.77 [0.56, 0.89] | 0.14 [-0.12, 0.40] | NA | NA |
| **SS** | 0.38 [-0.22, 0.87] | NA | 0.02 [-0.58, 0.61] | NA |
| **WD** | 0.71 [0.34, 0.94] | NA | 0.24 [-0.38, 0.78] | NA |
| **WS** | 0.55 [0.15, 0.86] | NA | NA | NA |
|  |  |  |  |  |
| **Species** | $r_{\Phi_{j}\psi}^{1} (type 1)$ | $r_{\Phi_{j}\psi}^{1} (type 2)$ | $r_{\Phi_{j}\pi} (type1)$ | $\begin{aligned} r_{\Phi_{j}\pi} \left( type2 \right) \end{aligned}$ |
| **AF** | 0.07 [-0.27,0.40] | 0.24 [-0.09, 0.59] | -0.12 [-0.50, 0.25] | 0.15 [-0.21, 0.53] |
| **BBA** | 0.43 [-0.05, 0.83] | 0.22 [-0.28, 0.67] | -0.11 [-0.54, 0.30] | -0.13 [-0.57, 0.33] |
| **BS** | 0.65 [0.25, 0.88] | 0.48 [0.09, 0.81] | 0.73 [0.37, 0.92] | 0.46 [0.06, 0.81] |
| **BT** | NA | NA | -0.18 [-0.59, 0.24] | 0.16 [-0.24, 0.58] |
| **EO** | 0.23 [-0.13, 0.68] | -0.02 [-0.55, 0.62] | 0.03 [-0.35, 0.41] | -0.03 [-0.45, 0.43] |
| **GS** | 0.22 [-0.42, 0.80] | 0.19 [-0.48, 0.77] | 0.25 [-0.37, 0.83] | 0.23 [-0.39, 0.79] |
| **GT** | NA | NA | -0.18 [-0.45, 0.10] | 0.09 [-0.21, 0.37] |
| **HS** | NA | NA | -0.05 [-0.53, 0.44] | 0.07 [-0.40, 0.60] |
| **Ki** | 0.09 [-0.22, 0.40] | 0.01 [-0.31, 0.32] | -0.07 [-0.38, 0.24] | 0.19 [-0.12, 0.50] |
| **MG** | 0.01 [-0.81, 0.73] | 0.11 [-0.60, 0.83] | -0.04 [-0.67, 0.60] | 0.07 [-0.66, 0.79] |
| **RD** | 0.22 [-0.40,0.80] | 0.36 [-0.26,0.86] | NA | NA |
| **SP** | 0.05 [-0.22, 0.34] | -0.23 [-0.50, 0.10] | 0.13 [-0.16, 0.41] | 0.04 [-0.23, 0.34] |
| **SS** | NA | NA | -0.38 [-0.86, 0.29] | 0.19 [-0.43, 0.77] |
| **WD** | NA | NA | -0.16 [-0.79, 0.56] | 0.18 [-0.30, 0.65] |
| **WS** | 0.29 [-0.04, 0.59] | 0.34 [0.01, 0.61] | NA | NA |
|  |  |  |  |  |
| **Species** | $\begin{aligned} r_{\Phi_{j}\Omega} \left( type 1 \right) \end{aligned}$ | $\begin{aligned} r_{\Phi_{j}\Omega} \left( type 2 \right) \end{aligned}$ | $r_{\Phi_{ad}\psi}^{1} (type 1)$ | $r_{\Phi_{ad}\psi}^{1} (type 2)$ |
| **AF** | NA | NA | 0.31 [-0.13, 0.69] | -0.13 [-0.51, 0.29] |
| **BBA** | NA | NA | -0.13 [-0.59, 0.34] | -0.04 [-0.50, 0.41] |
| **BS** | NA | NA | -0.09 [-0.44, 0.27] | 0.13 [-0.24, 0.52] |
| **BT** | 0.00 [-0.38, 0.37] | 0.32 [-0.10, 0.70] | NA | NA |
| **EO** | 0.09 [-0.31, 0.51] | 0.13 [-0.32, 0.55] | -0.20 [-0.55, 0.13] | 0.36 [0.00, 0.85] |
| **GS** | 0.04 [-0.63, 0.69] | 0.04 [-0.61, 0.66] | 0.12 [-0.57, 0.77] | -0.10 [-0.73, 0.63] |
| **GT** | -0.27 [-0.48, -0.02] | 0.33 [0.12, 0.53] | NA | NA |
| **HS** | -0.08 [-0.68, 0.56] | 0.10 [-0.51, 0.71] | NA | NA |
| **Ki** | NA | NA | -0.01 [-0.32, 0.29] | 0.10 [-0.22, 0.41] |
| **MG** | NA | NA | 0.40 [-0.24, 0.87] | 0.34 [-0.34, 0.89] |
| **RD** | 0.57 [0.15,0.88] | 0.62 [0.12,0.90] | 0.31 [-0.24,0.79] | 0.48 [-0.08,0.85] |
| **SP** | NA | NA | 0.02 [-0.26, 0.30] | -0.09 [-0.39, 0.23] |
| **SS** | -0.22 [-0.72, 0.29] | 0.37 [-0.18, 0.84] | NA | NA |
| **WD** | 0.02 [-0.71, 0.74] | 0.40 [-0.31, 0.89] | NA | NA |
| **WS** | NA | NA | 0.00 [-0.41, 0.37] | 0.07 [-0.33, 0.44] |
|  |  |  |  |  |
| **Species** | $r_{\Phi_{ad}\pi} \left( type 1 \right)$ | $r_{\Phi_{ad}\pi} \left( type 2 \right)$ | $r_{\Phi_{ad}\Omega} \left( type 1 \right)$ | $r_{\Phi_{ad}\Omega} \left( type 2 \right)$ |
| **AF** | 0.38 [-0.07, 0.82] | 0.12 [-0.31, 0.52] | NA | NA |
| **BBA** | -0.10 [-0.53, 0.34] | -0.07 [-0.50, 0.36] | NA | NA |
| **BS** | 0.23 [-0.16, 0.56] | 0.32 [-0.06, 0.68] | NA | NA |
| **BT** | 0.16 [-0.33, 0.65] | 0.04 [-0.47,0.52] | 0.02 [-0.41, 0.47] | -0.20 [-0.63, 0.27] |
| **EO** | -0.14 [-0.49, 0.26] | 0.11 [-0.31, 0.52] | -0.11 [-0.56, 0.34] | -0.07 [-0.54, 0.43] |
| **GS** | 0.17 [-0.52, 0.78] | 0.13 [-0.53, 0.52] | -0.05 [-0.70, 0.63] | -0.01 [-0.64, 0.61] |
| **GT** | -0.25 [-0.54, 0.05] | 0.02 [-0.27, 0.30] | -0.40 [-0.60, -0.15] | 0.10 [-0.15, 0.34] |
| **HS** | 0.30 [-0.21, 0.78] | 0.18 [-0.30, 0.64] | 0.03 [-0.61, 0.68] | 0.00 [-0.63, 0.62] |
| **Ki** | -0.27 [-0.55, 0.04] | -0.31 [-0.60, 0.01] | NA | NA |
| **MG** | 0.13 [-0.46, 0.73] | 0.36 [-0.27, 0.85] | NA | NA |
| **RD** | NA | NA | 0.53 [0.13,0.87] | 0.60 [0.24,0.85] |
| **SP** | 0.00 [-0.29, 0.29] | 0.10 [-0.16, 0.39] | NA | NA |
| **SS** | -0.17 [-0.73, 0.45] | 0.23 [-0.41, 0.76] | -0.29 [-0.76, 0.25] | 0.37 [-0.17, 0.81] |
| **WD** | -0.08 [-0.67, 0.57] | 0.25 [-0.13, 0.60] | 0.01 [-0.67, 0.66] | 0.37 [-0.24, 0.82] |
| **WS** | NA | NA | NA | NA |

**Table S3**: Relationships between the correlation pair of demographic parameters and generation time (a metric measuring the position of the species along the slow-fast continuum). Values include the number of species for which the relationship was assessed (n), the estimated slope of the regression, the 95% credible interval of the slope (95% CRI), the proportion of the posterior distribution that had the same sign as the slope (P) and the proportion of among-species variance in correlation explained by generation time (R2). For the correlation between survival and reproductive success, we estimated correlations between reproductive success and both survival from the previous reproductive season (type 1) and to the next (type 2), see Fig. 1. For model notation, “$\Phi$” indicates survival, “$\psi$” breeding probability, “$\pi$” breeding success, “$\Omega$” productivity, “*j*” juvenile, and “*ad*” adult.

| **Correlation pair** | **n** | **Slope** | **95% CRI** | **P** | **R2** |
| --- | --- | --- | --- | --- | --- |
| $r_{\Phi_{j}\Phi_{ad}}$ | 15 | 0.000 | [-0.021,0.022] | 0.51 | 0.00 |
| $r_{\Phi_{j}\psi}$ (type 1) | 10 | -0.007 | [-0.028,0.015] | 0.76 | 0.08 |
| $r_{\Phi_{j}\psi}$ (type 2) | 10 | -0.011 | [-0.031,0.011] | 0.88 | 0.18 |
| $r_{\Phi_{ad}\psi}$ (type 1) | 10 | -0.011 | [-0.035,0.010] | 0.88 | 0.15 |
| $r_{\Phi_{ad}\psi}$ (type 2) | 10 | -0.006 | [-0.027,0.015] | 0.74 | 0.07 |
| $r_{\Phi_{j}\pi}$ (type 1) | 13 | 0.002 | [-0.020,0.023] | 0.58 | 0.00 |
| $r_{\Phi_{j}\pi}$ (type 2) | 13 | -0.008 | [-0.018,0.001] | 0.96 | 0.27 |
| $r_{\Phi_{ad}\pi}$ (type 1) | 13 | -0.000 | [-0.016,0.018] | 0.50 | 0.00 |
| $r_{\Phi_{ad}\pi}$ (type 2) | 13 | -0.005 | [-0.018,0.008] | 0.79 | 0.06 |
| $r_{\Phi_{j}\Omega}$ (type 1) | 9 | 0.009 | [-0.035,0.050] | 0.69 | 0.04 |
| $r_{\Phi_{j}\Omega}$ (type 2) | 9 | -0.007 | [-0.036,0.021] | 0.71 | 0.07 |
| $r_{\Phi_{ad}\Omega}$ (type 1) | 9 | 0.001 | [-0.048,0.059] | 0.54 | 0.01 |
| $r_{\Phi_{ad}\Omega}$ (type 2) | 9 | -0.010 | [-0.053,0.031] | 0.71 | 0.07 |
| $r_{\psi\pi}$ | 9 | -0.007 | [-0.039,0.022] | 0.72 | 0.07 |
| $r_{\pi\Omega}$ | 8 | 0.012 | [-0.014,0.039] | 0.85 | 0.22 |

**Fig S1**: Effect of the prior on correlation estimates for the golden-mantled ground squirrel population. We compared the estimates obtained in this paper (prior 1) with estimates obtained using two new priors (prior 2 & 3, see appendix S3). Demographic parameters include juvenile survival ($\Phi_{j}$), adult survival ($\Phi_{ad}$), breeding probability ($\psi$), reproductive success ($\pi$) and productivity ($\Omega$). Here we estimated correlations between reproductive performance and survival from the previous reproductive season.


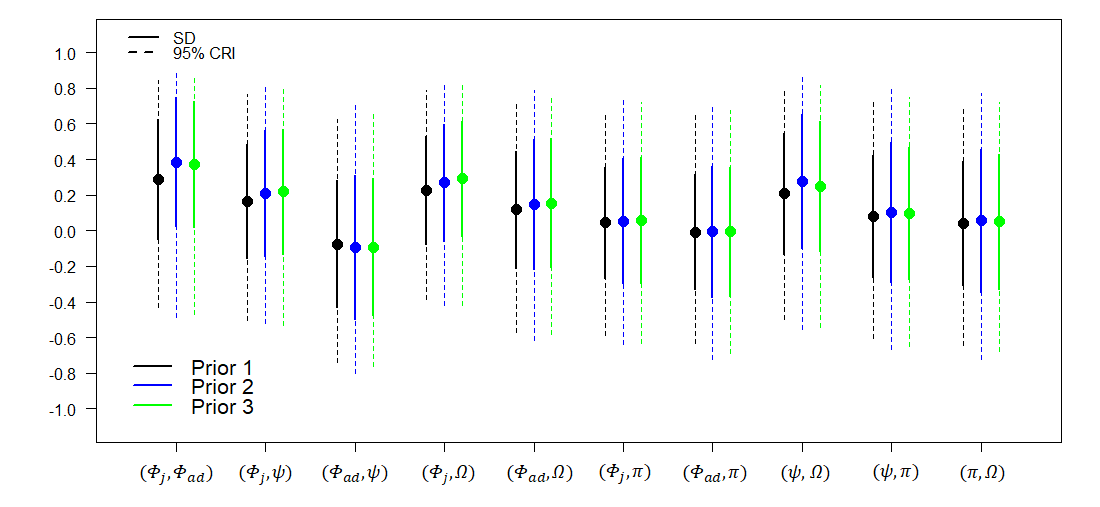


**Fig. S2**: Temporal correlations estimated for three pairs of demographic parameters including juvenile survival ($\Phi_{j}$), adult survival ($\Phi_{ad}$) and reproductive success ($\pi$) when population density was accounted for (orange) or not (black). We estimated correlations between reproductive success and both survival to the next (blue) and from the previous reproductive season (green). Species names: AF = Antarctic fulmar, BBA = black-browed albatross, BS = bighorn sheep, BT = blue tit, EO = Eurasian oystercatcher, GS = golden-mantled ground squirrel, GT = great tit, HS = house sparrow, Ki = kittiwake, MG = mountain goat, RD = roe deer, SS = Savannah sparrow, WD = white-throated dipper, WS = Weddell seal. For notation, “SD” indicates standard deviation, “CRI” indicates credible interval and “P” indicates the proportion of the posterior distribution that has the same sign as the mean effect size.


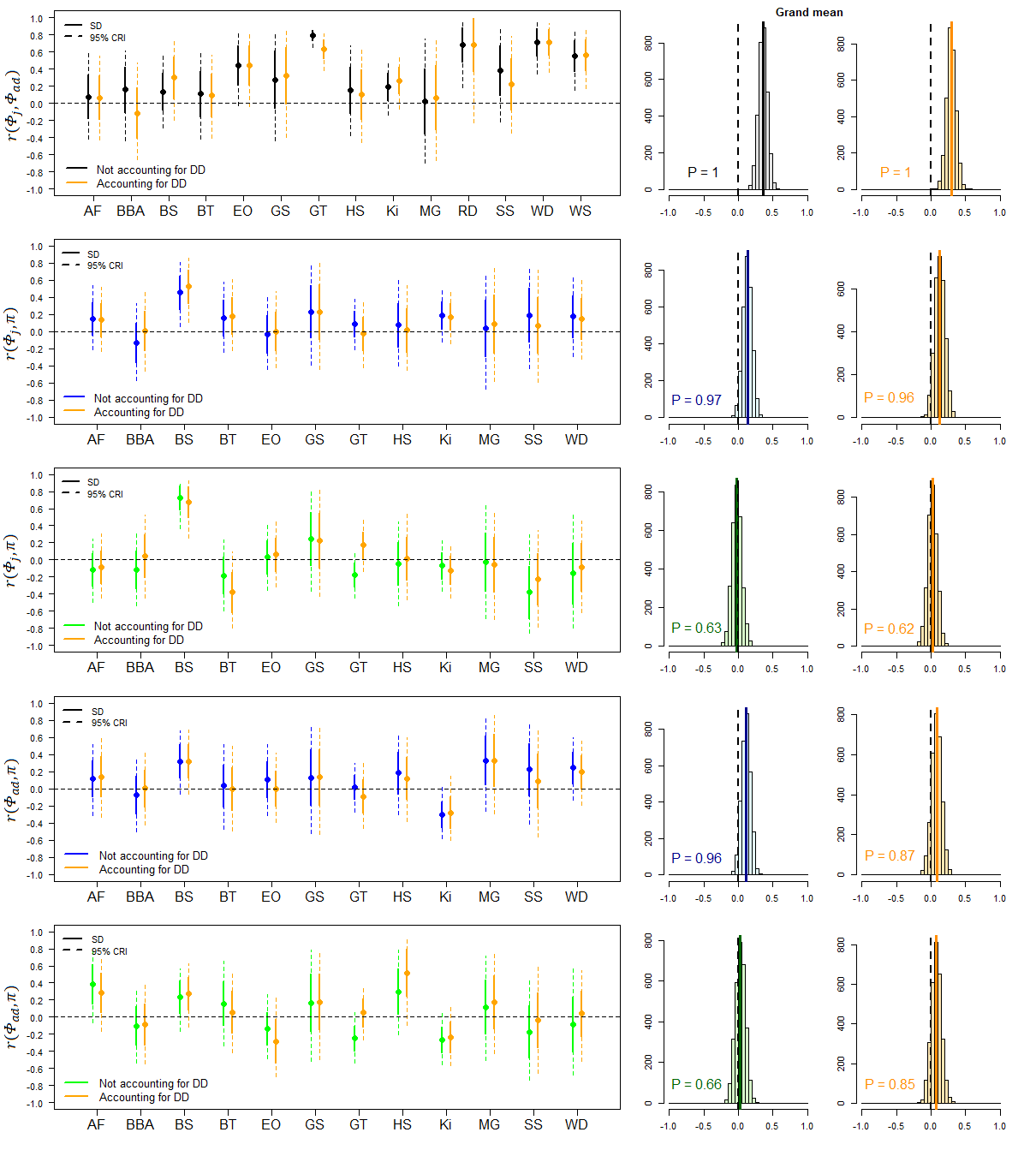


**Fig. S3**: Temporal correlations estimated for three pairs of demographic parameters including reproductive probability ($\psi$) and reproductive success ($\pi$) and productivity ($\Omega$) when population density was accounted for (orange) or not (black). Species names: AF = Antarctic fulmar, BBA = black-browed albatross, BS = bighorn sheep, BT = blue tit, EO = Eurasian oystercatcher, GS = golden-mantled ground squirrel, GT = great tit, HS = house sparrow, Ki = kittiwake, MG = mountain goat, RD = roe deer, SS = Savannah sparrow, WD = white-throated dipper. For notation, “SD” indicates standard deviation, “CRI” indicates credible interval and “P” indicates the proportion of the posterior distribution that has the same sign as the mean effect size.


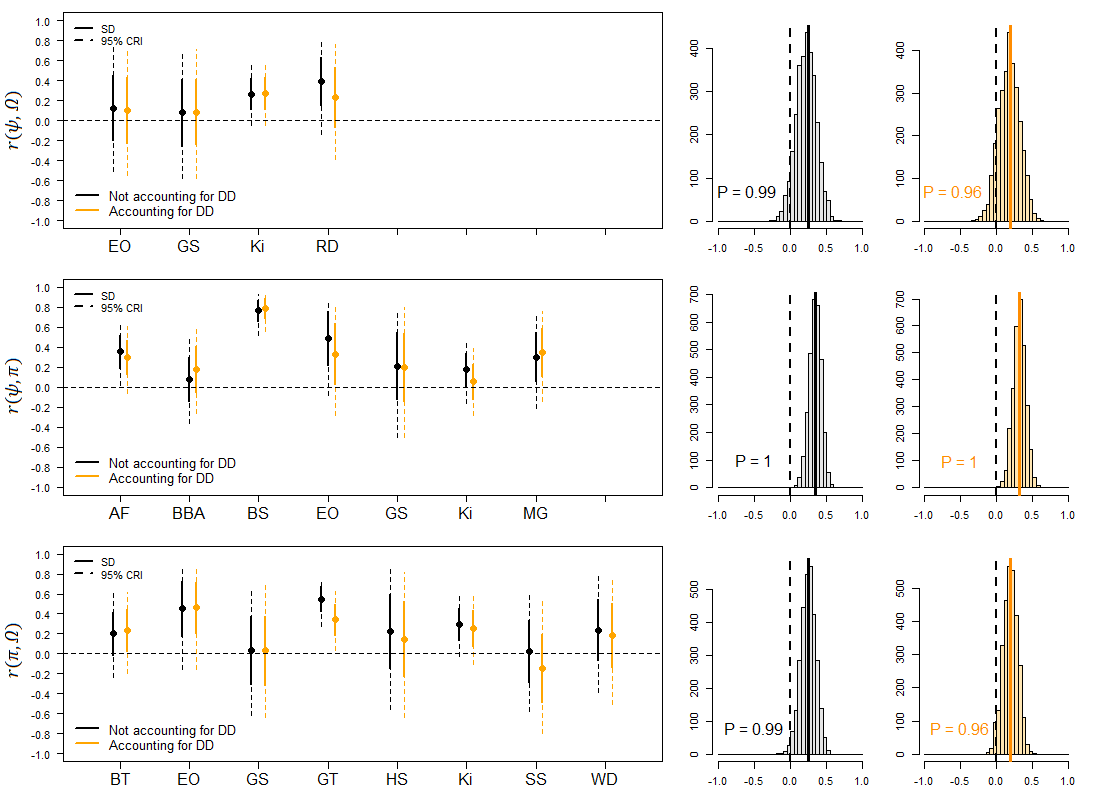


**Fig. S4**: Temporal correlations estimated for three pairs of demographic parameters including juvenile survival ($\Phi_{j}$), adult survival ($\Phi_{ad}$) and breeding probability ($\psi$) when population density was accounted for (orange) or not (black). For correlation between survival and reproductive success, we estimated correlations between reproductive success and both survival to the next (blue) and from the previous reproductive season (green). Species names: AF = Antarctic fulmar, BBA = black-browed albatross, BS = bighorn sheep, EO = Eurasian oystercatcher, GS = golden-mantled ground squirrel, Ki = kittiwake, MG = mountain goat, RD = roe deer, WS = Weddell seal. For notation, “SD” indicates standard deviation, “CRI” indicates credible interval and “P” indicates the proportion of the posterior distribution that has the same sign as the mean effect size.


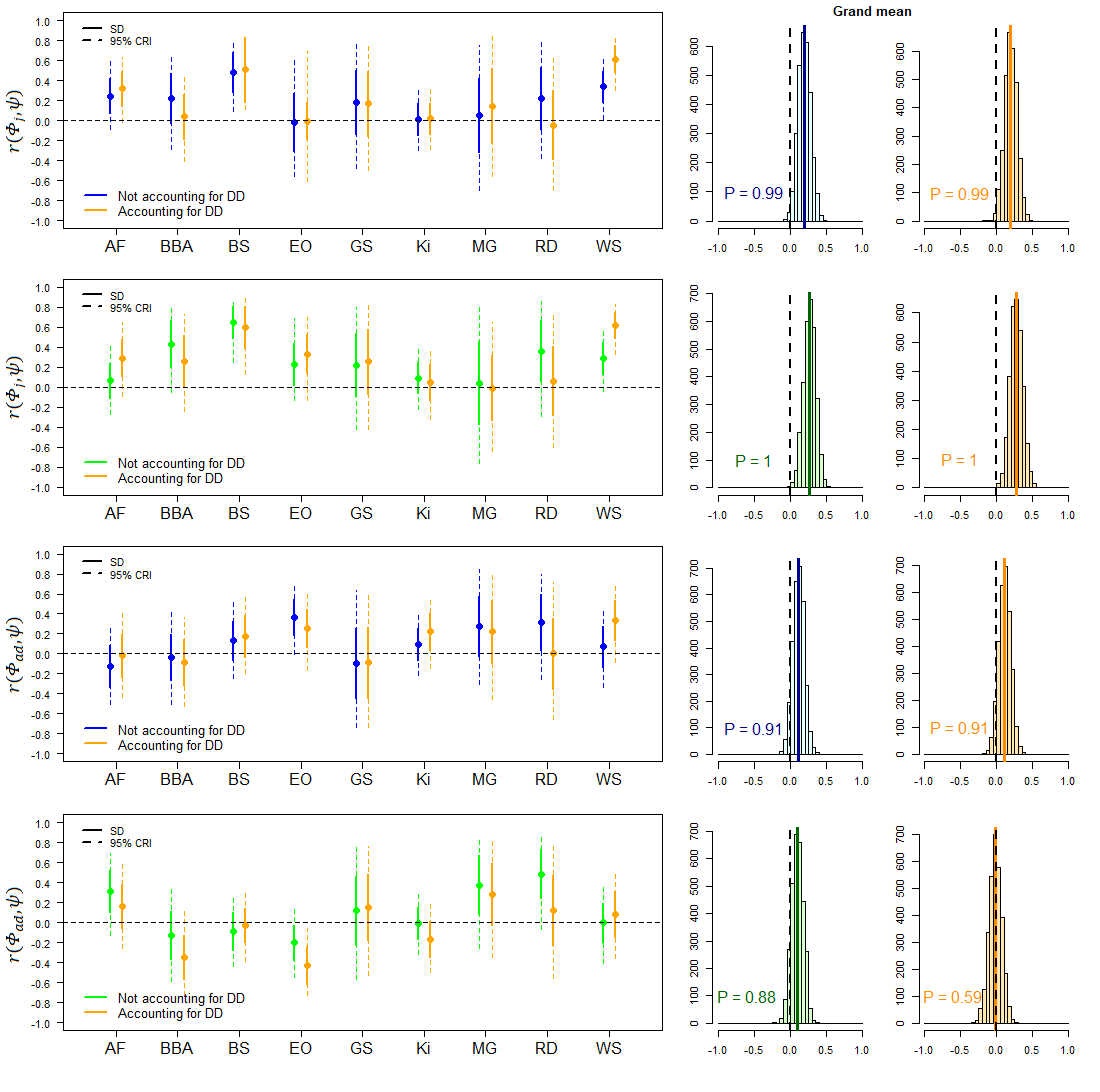


**Fig. S5**: Temporal correlations estimated for three pairs of demographic parameters including juvenile survival ($\Phi_{j}$), adult survival ($\Phi_{ad}$) and productivity ($\Omega$) when population density was accounted for (orange) or not (black). We estimated correlations between productivity and both survival to the next (blue) and from the previous reproductive season (green). Species names: BT = blue tit, EO = Eurasian oystercatcher, GS = golden-mantled ground squirrel, GT = great tit, HS = house sparrow, Ki = kittiwake, SS = Savannah sparrow, WD = white-throated dipper. For notation, “SD” indicates standard deviation, “CRI” indicates credible interval and “P” indicates the proportion of the posterior distribution that has the same sign as the mean effect size.


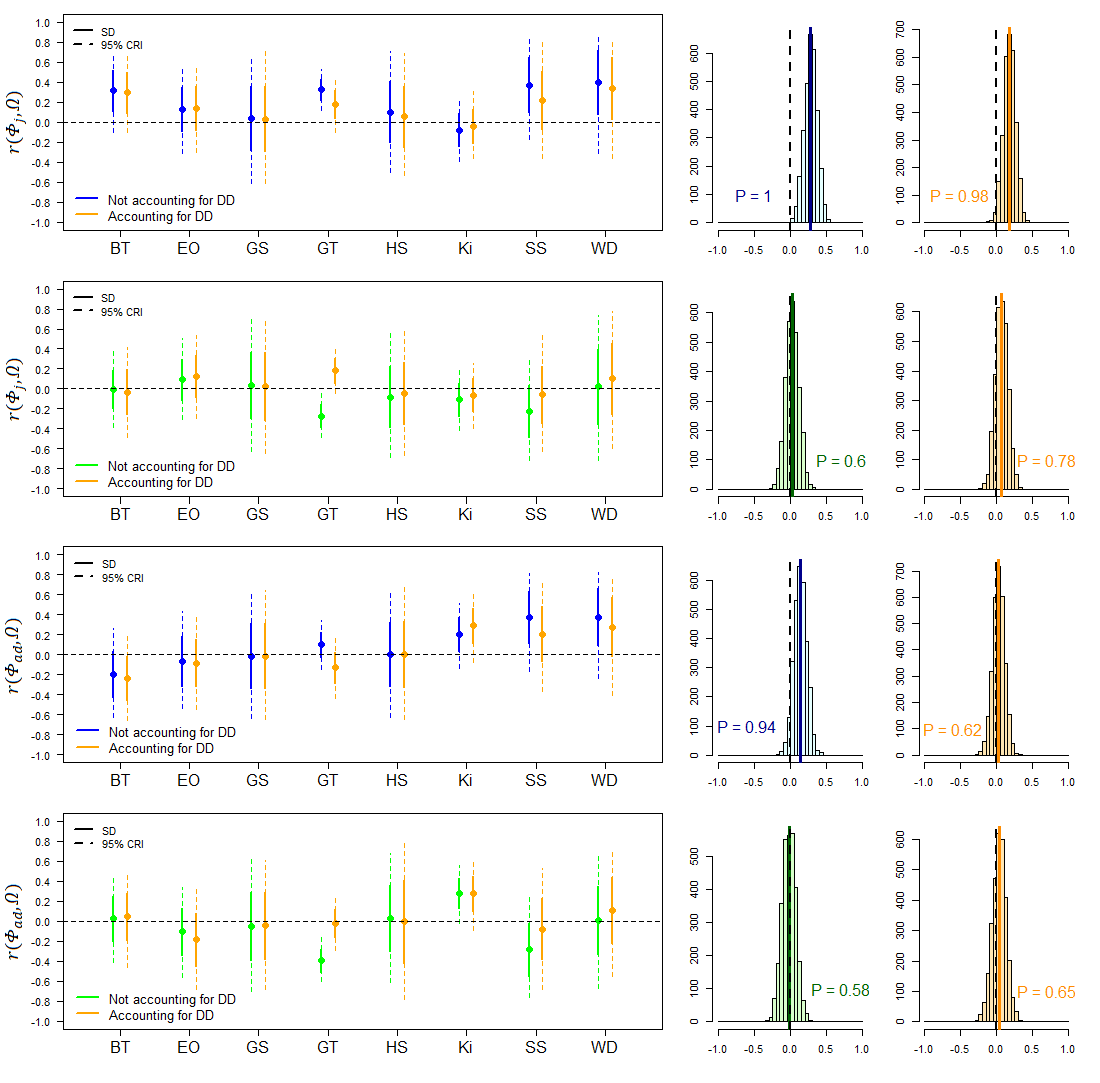


**Appendix S1**: JAGS code used to estimate the mean, variance and covariance of survival and reproductive parameters for the white-throated dipper. In this example, the parametrization allows estimation of the correlation between reproductive performances and survival from the previous breeding season.

model {

### LIKELIHOOD ###

for(i in 1:n.ind) {

## First capture

# Breeding success

SUCCESS[i, FIRST[i]] ~ dbern(ilogit(mu.psi[AGE[i,FIRST[i]]])) + epsilon[FIRST[i],3]))

# Productivity

OFFSPRING[i, FIRST[i]] ~ dnorm(pi[i, FIRST[i]] * SUCCESS[i,FIRST[i]], tau.off) T(SUCCESS[i, FIRST[i]],)

pi[i, FIRST[i]] <- mu.pi[AGE[i,FIRST[i]]] + epsilon[FIRST[i],4]

## Rest of the life histories

for(t in (FIRST[i] + 1):LAST[i]) {

# survival

ALIVE[i, t] ~ dbern(surv[i, t] * ALIVE[i, t-1])

surv[i, t] <- ilogit(mu.phi[AGE[i,t-1]] + ifelse(AGE[i,t-1] == 1, epsilon[t, 1], epsilon[t, 2]))

# Breeding success

SUCCESS[i, t] ~ dbern(psi[i, t] * ALIVE[i, t])

psi[i, t] <- ilogit(mu.psi[AGE[i,t]] + epsilon[t, 3])

# Productivity

OFFSPRING[i, t] ~ dnorm(pi[i, t] * SUCCESS[i, t], tau.off) T(SUCCESS[i,t],)

pi[i, t] <- mu.pi[AGE[i,t]] + epsilon[t, 4]

# detection

DETECTED[i, t] ~ dbern(proba_star[i, t] * ALIVE[i, t])

logit(proba_star[i, t]) <- mu.p[AGE[i,t]]

}

}

### PRIORS ###

# survival

mu.phi[1] ~ dnorm(0.0, 0.4444444)

mu.phi[2] ~ dnorm(0.0, 0.4444444)

mu.phi[3] <- mu.phi[2]

# Nest success

mu.psi[1] ~ dnorm(0.0, 0.4444444)

mu.psi[2] ~ dnorm(0.0, 0.4444444)

mu.psi[3] ~ dnorm(0.0, 0.4444444)

# Productivity

mu.pi[1] ~ dnorm(0.0, 0.4444444)

mu.pi[2] ~ dnorm(0.0, 0.4444444)

mu.pi[3] ~ dnorm(0.0, 0.4444444)

tau.off <- pow(sigma.off, -2)

sigma.off ~ dunif(0.001, 5)

# detection

mu.p[1] ~ dnorm(0.0, 0.4444444)

mu.p[2] ~ dnorm(0.0, 0.4444444)

mu.p[3] ~ dnorm(0.0, 0.4444444)

# Temporal random effects

for (j in 1:4) {

E[j, j] ~ dnorm(0.0, 0.4444444)T(0.0,)

DeltaE[j, j] <- 1/tauE[j]

tauE[j] ~ dgamma(1.5, 1.5)

LE[j, j] <- 1.0

}

for (j in 1:3) {

for (k in (j+1):4) {

LE[j, k] <- 0.0; E[j, k] <- 0.0; DeltaE[j, k] <- 0.0

LE[k, j] ~ dnorm(0.0, 4.0); E[k, j] <- 0.0; DeltaE[k, j] <- 0.0

}

}

# covariance matrix

Lambda <- E %*% LE %*% DeltaE %*% t(LE) %*% E

for(i in 1:n.year){

epsilon[i, 1] <- E[1, 1] * (LE[1, 1] * xi_e[i, 1])

epsilon[i, 2] <- E[2, 2] * (LE[2, 1] * xi_e[i, 1] + LE[2, 2] * xi_e[i, 2])

epsilon[i, 3] <- E[3, 3] * (LE[3, 1] * xi_e[i, 1] + LE[3, 2] * xi_e[i, 2] + LE[3, 3] * xi_e[i, 3])

epsilon[i, 4] <- E[4, 4] * (LE[4, 1] * xi_e[i, 1] + LE[4, 2] * xi_e[i, 2] + LE[4, 3] * xi_e[i, 3] + LE[4, 4] * xi_e[i, 4])

for(j in 1:4){

xi_e[i, j] ~ dnorm(0.0, tauE[j])

}

}

# Derived quantities

mean.phi[1] <- ilogit(mu.phi[1])

mean.phi[2] <- ilogit(mu.phi[2])

mean.psi[1] <- ilogit(mu.psi[2])

mean.psi[2] <- ilogit(mu.psi[3])

mean.pi[1] <- mu.pi[2]

mean.pi[2] <- mu.pi[3]

mean.p[1] <- ilogit(mu.p[2])

mean.p[2] <- ilogit(mu.p[3])

sigma.phi1 <- sqrt(Lambda[1, 1])

sigma.phi2 <- sqrt(Lambda[2, 2])

sigma.psi <- sqrt(Lambda[3, 3])

sigma.pi <- sqrt(Lambda[4, 4])

cor.phi1.phi2 <- Lambda[1, 2] / sqrt(Lambda[1, 1] * Lambda[2, 2])

cor.phi1.psi <- Lambda[1, 3] / sqrt(Lambda[1, 1] * Lambda[3, 3])

cor.phi1.pi <- Lambda[1, 4] / sqrt(Lambda[1, 1] * Lambda[4, 4])

cor.phi2.psi <- Lambda[2, 3] / sqrt(Lambda[2, 2] * Lambda[3, 3])

cor.phi2.pi <- Lambda[2, 4] / sqrt(Lambda[2, 2] * Lambda[4, 4])

cor.psi.pi <- Lambda[3, 4] / sqrt(Lambda[3, 3] * Lambda[4, 4])

}

**Appendix S2:** Details about the modelling of the variance-covariance matrix using the Cholesky decomposition with Parameter Expansion.

Let $y_{it}^{j}$ denotes the $j^{th}$ response variable (a measurement) on individual $i$ in year $t$. Consider analyzing these data with a multivariate linear mixed model (dropping the $j$ superscript for legibility):

$$y_{it}^{j}= \mathbf{X}_{it}^{'}\boldsymbol{\beta}+\mathbf{Z}_{it}^{'}\mathbf{b}_{i}+ \varepsilon_{it} , \varepsilon_{it} \sim N(0,\sigma^{2})$$

where $\mathbf{X}$ and $\mathbf{Z}$ are design matrices; and $\boldsymbol{\beta}$ is a vector of $p$ fixed effects and $\mathbf{b}_{i}$ a vector (of length $m$) of random effects:

$$\boldsymbol{b}_{i} \sim N(0_{m},\boldsymbol{\Omega})$$

$\boldsymbol{\Omega}$ is a covariance matrix of dimensions $m\times m$. The covariance matrix is a symmetric, positive-definite matrix with variance parameters on the diagonal and covariance parameters elsewhere. The requirement of the matrix to be positive-definite means that simply bounding the correlation parameters to lie between $-1$ and $1$ is not enough as there are additional constraints to satisfy (Budden et al. 2007). In order to do so, several authors have proposed different strategies. Chen and Dunson (2003) proposed to decompose $\boldsymbol{\Omega}$ into a diagonal $m\times m$ matrix $\mathbf{D}$ and a lower triangular $m\times m$ matrix $\mathbf{L}$ with $1$s on the diagonal and $0$s above the diagonal:

$$\boldsymbol{\Omega}=\mathbf{DL}\left( \mathbf{DL} \right)^{'}=\mathbf{DLL}'\mathbf{D}$$

The first equation above can then be rewritten as:

$$y_{it}= \mathbf{X}_{it}^{'}\boldsymbol{\beta}+\mathbf{Z}_{it}^{'}\mathbf{DLu}_{i}+ \varepsilon_{it} , \varepsilon_{it} \sim N(0,\sigma^{2})$$

where $\mathbf{u}_{i}$ is a vector of standardized normal variates. This equation is easier to work with as the random effects $\mathbf{b}_{i}$ can be expressed as linear regression coefficients conditional on $\mathbf{D}$, $\mathbf{L}$ and $\mathbf{u}_{i}$ (Chen and Dunson 2003). For example, with $m=2$, that is a bivariate random effect $\mathbf{b}_{i}=\left( b_{i1}, b_{i2} \right)$,

$$\left\{ \begin{matrix} b_{i1}= d_{1}u_{i1} , u_{i1} \sim N(0,1) \\ b_{i2}= d_{2}\left( {l_{21}u}_{i1}+ u_{i2} \right) ,u_{i2} \sim N(0,1) \end{matrix} \right.$$

with $\mathbf{D}=\left[ \begin{matrix} d_{1} & 0 \\ 0 & d_{2} \end{matrix} \right]$ and $\mathbf{L}=\left[ \begin{matrix} 1 & 0 \\ l_{21} & 1 \end{matrix} \right]$. $N\left( \mu,\sigma^{2} \right)$ denotes a normal distribution of location parameter $\mu$ and scale parameter $\sigma$.

This equation is useful because the covariance matrix is never formed explicitly and for an MCMC algorithm, sampling will be more efficient as the sampler may be able to explore more easily the parameter space (i.e., the autocorrelation between draws/iterations will be reduced). This is because unconstrained priors can be used on the elements of $\mathbf{D}$ and $\mathbf{L}$, and the resulting matrix will be a valid covariance matrix.

To further improve computational efficiency and reduce dependence among MCMC draws, Kinney & Dunson (2008, chapter 3 in *Random Effects and Latent Variable Model Selection*) proposed the following parameter expansion:

$$y_{it}= \mathbf{X}_{it}^{'}\boldsymbol{\beta}+\mathbf{Z}_{it}^{'}{\mathbf{AL}\xi}_{i}+ \varepsilon_{it} , \varepsilon_{it} \sim N(0,\sigma^{2})$$

where $\xi_{i}\sim N\left( \mathbf{0}_{m},\boldsymbol{\Delta} \right)$, $\boldsymbol{\Delta}=diag\left( \delta_{1},...,\delta_{m} \right)$ and $\mathbf{A}=diag\left( \alpha_{1},...,\alpha_{m} \right)$ are diagonal $m\times m$ matrices; and $\mathbf{L}$ is a lower triangular $m\times m$ matrix with $1$s on the diagonal and $0$s above the diagonal. With this parametrization, the covariance matrix is:

$$\boldsymbol{\Omega}=\boldsymbol{AL\Delta L}'\mathbf{A}$$

The random effects $\mathbf{b}_{i}$ can again be expressed as linear regression coefficients conditional on $\boldsymbol{\Delta}$, $\mathbf{A}$, $\mathbf{L}$ and $\xi_{i}$. For example, with $m=2$, that is a bivariate random effect $\mathbf{b}_{i}=\left( b_{i1}, b_{i2} \right)$,

$$\left\{ \begin{matrix} b_{i1}= \alpha_{1}\xi_{i1} , \xi_{i1} \sim N(0,\delta_{1}^{2}) \\ b_{i2}= \alpha_{2}\left( {l_{21}\xi}_{i1}+ \xi_{i2} \right) ,\xi_{i2} \sim N(0,\delta_{2}^{2}) \end{matrix} \right.$$

with $\boldsymbol{\Delta}=\left[ \begin{matrix} \delta_{1} & 0 \\ 0 & \delta_{2} \end{matrix} \right]$, $\mathbf{A}=\left[ \begin{matrix} \alpha_{1} & 0 \\ 0 & \alpha_{2} \end{matrix} \right]$ and $\mathbf{L}=\left[ \begin{matrix} 1 & 0 \\ l_{21} & 1 \end{matrix} \right]$.

To induce marginal half-t priors $\mathcal{T}^{+}\left( 0,a^{2},\nu\right)$ (where $a$ is a scale parameter and $\nu$ denotes the degree of freedom of the Student-t distribution) on the standard deviations of the random effects (assuming that they are uncorrelated, that is all Cholesky factors $l_{{qr}_{q>r}}=0)$, the following priors can be used:

$\alpha\sim N^{+}\left( 0,a^{2} \right)$ where $a$ is the prior scale, and $\delta^{2}\sim\Gamma^{-1}\left( \frac{\nu}{2},\frac{\nu}{2} \right)$ where $\Gamma^{-1}$ denotes the Inverse Gamma distribution (shape and rate parametrization). We set $\nu=3$. Chen & Dunson (2003) further proposed to set $a=1.5$. The same authors use a normal prior on the elements of $\mathbf{L}$, $l_{{qr}_{q>r}}\mathcal{\sim N}\left( 0,b^{2} \right)$ with $b=0.5$. See appendix S3 for a graphical representation of these priors.

**Appendix S3**: Effect of the prior used for the variance covariance matrix on correlation estimates.

To assess the effect of the chosen prior (prior 1) on correlation estimates, we re-ran models using two new priors (prior 2 & 3). We ran the model for the golden-mantled ground squirrel population, one of the smallest datasets in this study regarding both the number of individuals and the number of monitoring years. This allows us to assess the influence of the prior where its potential effect is the largest. New priors were modified versions of the initial prior used in this study (see appendix S2). For the first new prior (prior 2), we modified the parameter *b* from 0.5 to 0.7. For the second new prior (prior 3), we modified both parameters *b* from 0.5 to 0.7 and *ν* from 3 to 8. These parametrizations give different shapes of prior for correlation with only minor modification for standard deviation of the random effects, as illustrated bellow:


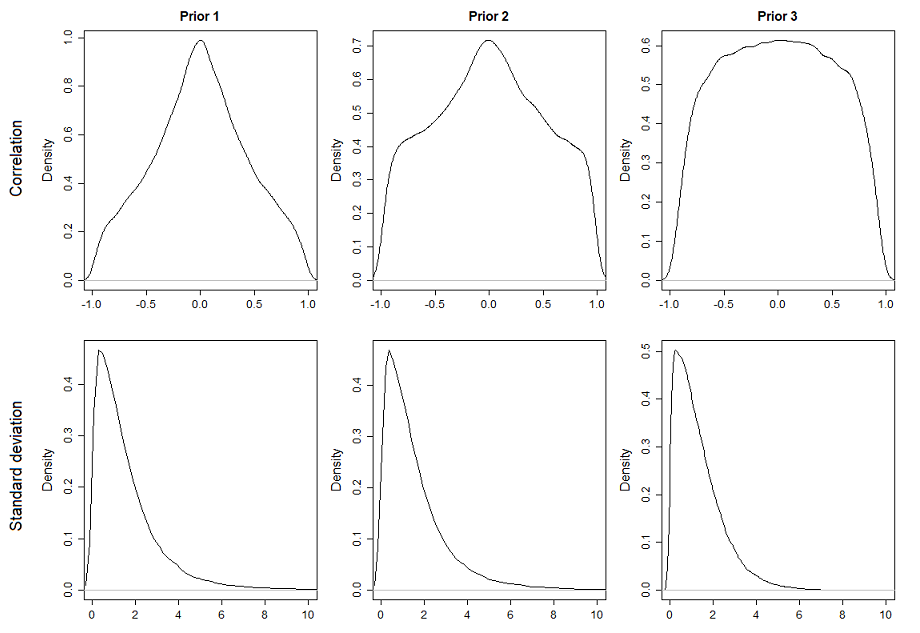


**Appendix S4**: Accounting for population density

Population density (N) was estimated either directly as the number of breeding females (e.g., Weddell seal), the number of active nests (e.g., dipper) or the total number of adults (e.g., house sparrow), or indirectly as the proportion of occupied nest boxes (e.g, blue tit). To account for population density, we included a linear effect of N in all equations modelling demographic parameters. For instance, to estimate juvenile survival accounting for population density, we use the following equation:

$\mathrm{Alive}_{i,t} \sim Bernoulli \left( {logit}^{-1}\left( \mu_{\Phi}+ \beta_{\Phi,juv}*N[t-1] + \alpha_{t,\Phi,juv} \right) \right)$

where $\mu_{\Phi}$ is the intercept on the logit scale, $\beta_{\Phi,juv}$ is the slope quantifying the effect of population density in the year t-1 and $\alpha_{t,\Phi,juv}$ is the temporal random effect.

For breeding parameters, we considered the effect of current population density (i.e., N[t] instead of N[t-1]). For instance, conditional on being alive, individual *i* may breed as:

$$\left( \mathrm{Breed}_{i,t}|\mathrm{Alive}_{i,t}=1 \right) \sim Bernoulli \left( {logit}^{-1}\left( \mu_{\psi}+f_{\psi}\left( {age}_{i,t} \right)+ \gamma_{\psi}*{BS}_{i,t-1}+ \beta_{\psi}*N\left[ t \right]+ \alpha_{t,\psi} \right) \right)$$

where ${Breed}_{i,t}$ is a dummy variable indicating whether individual *i* bred in year *t*, $\mu_{\psi}$ is the intercept on logit scale, $f_{\psi}\left( {age}_{i,t} \right)$ is a function of age, $\gamma_{\psi}$­ is the the effect of the breeding state, $\beta_{\psi}$ is the slope quantifying the effect of population density the year t and $\alpha_{t,\psi}$ is the temporal random effect.

For the prior of β parameters, we used a uniform distribution on the range [-5,5]. The other parts of the model, including the modelling of detection and temporal variance and covariance, were unchanged.
